# Maternal biomarker patterns for metabolism and inflammation in pregnancy are influenced by multiple micronutrient supplementation and associated with child biomarker patterns and nutritional status at 9-12 years of age

**DOI:** 10.1371/journal.pone.0216848

**Published:** 2020-08-07

**Authors:** Lidwina Priliani, Sukma Oktavianthi, Elizabeth L. Prado, Safarina G. Malik, Anuraj H. Shankar

**Affiliations:** 1 Eijkman Institute for Molecular Biology, Ministry of Research and Technology/National Research and Innovation Agency, Jakarta, Indonesia; 2 Summit Institute of Development, Mataram, Lombok, West Nusa Tenggara, Indonesia; 3 Department of Nutrition, University California at Davis, Davis, California, United States of America; 4 Eijkman-Oxford Clinical Research Unit, Eijkman Institute for Molecular Biology, Jakarta, Indonesia; 5 Nuffield Department of Medicine, Centre for Tropical Medicine and Global Health, University of Oxford, Oxford, United Kingdom; PLOS ONE, UNITED STATES

## Abstract

Maternal nutritional status influences fetal development and long-term risk for adult non-communicable diseases. However, the underlying mechanisms remain poorly understood. We examined whether biomarkers for metabolism and inflammation during pregnancy were associated with maternal health and with child biomarkers and health at 9–12 years of age in 44 maternal-child dyads from the Supplementation with Multiple Micronutrients Intervention Trial (SUMMIT, ISRCTN34151616) in Lombok, Indonesia. Archived blood for each dyad from maternal enrollment, later in pregnancy, postpartum, and from children at 9–12 years comprised 132 specimens. Multiplex microbead immunoassays were used to quantify vitamin D-binding protein (D), adiponectin (A), retinol-binding protein 4 (R), C-reactive protein (C), and leptin (L). Principal component analysis (PCA) revealed distinct variance patterns, i.e. principal components (PC), for baseline pregnancy, bp.pc1.D↓A↓R↓ and bp.pc2.C↓L↑; combined follow-up during pregnancy and postpartum, dp-pp.pc1.D↑↓A↑R↑↓L↓ and dp-pp.pc2.A↑C↑L↑; and children, ch.pc1.D↑R↑C↑ and ch.pc2.D↓A↑L↑. Maternal multiple micronutrient (MMN) supplementation led to an association of baseline maternal bp.pc2.C↓L↑ with decreased post-supplementation maternal dp-pp.pc2.A↑C↑L↑ (*p* = 0.022), which was in turn associated with both increased child ch.pc1.D↑R↑C↑ (*p* = 0.036) and decreased child BMI z-score (BMIZ) (*p* = 0.022). Further analyses revealed an association between maternal dp-pp.pc1.D↑↓A↑R↑↓L↓ and increased child BMIZ (*p =* 0.036). Child ch.pc1.D↑R↑C↑ was associated with decreased birth weight (*p =* 0.036) and increased child BMIZ (*p* = 0.002). Child ch.pc2.D↓A↑L↑ was associated with increased child BMIZ (*p* = 0.005), decreased maternal height (*p =* 0.030) and girls (*p =* 0.002). A pattern of elevated maternal adiponectin and leptin in pregnancy was associated with increased C-reactive protein, vitamin A, and D binding proteins pattern in children, suggesting biomarkers acting in concert may have qualitative as well as quantitative influence beyond single biomarker effects. Patterns in pregnancy proximal to birth were more associated with child status. In addition, child patterns were more associated with child status, particularly child BMI. MMN supplementation affects maternal biomarker patterns of metabolism and inflammation in pregnancy, and potentially in the child. However, child nutrition conditions after birth may have a greater impact on metabolism and inflammation.

## Introduction

Emerging epidemiological evidence has shown that the risk for non-communicable diseases (NCDs) during childhood or as an adult is mediated in part by maternal nutrition in pregnancy and fetal growth [1–3]. Studies in animal models indicate that alterations in nutritional, metabolic, immune and hormonal milieu *in-utero* profoundly affect long-term health of the offspring, including increased risk for NCDs such as diabetes, obesity or cardiovascular disease [4,5]. Knowledge of the underlying mechanisms of these effects remains limited, although evidence is growing for the pivotal roles of metabolism-related hormones and inflammatory mediators [6,7].

Adipocytokines, including leptin, adiponectin, and retinol binding protein 4 (RBP4), play an important role in regulating metabolism, energy homeostasis and inflammatory responses [8–11]. Leptin is involved in body weight control by acting on the satiety center in the hypothalamus [12]. Leptin also promotes fetal growth and regulates fetal adipose tissue development [13]. Adiponectin plays a role in the catabolism of fatty acids and carbohydrates, improvement of insulin sensitivity and reduction of inflammation [14]. RBP4, previously thought to act as a specific transport protein for retinol, has been added to the family of adipocytokines given its role in obesity-induced insulin resistance [15]. Increased concentrations of both leptin and RBP4 have been associated with increased body mass index (BMI) [16,17], while adiponectin concentration was negatively associated with BMI [18]. Morevover, elevated concentrations of these adipocytokines during pregnancy have also been associated with adverse conditions, including gestational diabetes, preeclampsia and intrauterine growth restriction (IUGR) [19–22]. A previous study reported that maternal leptin and adiponectin concentrations were correlated with fetal leptin and adiponectin concentrations [23].

Inflammatory markers have been associated with increased risk of cardiovascular disease [24]. Specifically, higher C-reactive protein (CRP) concentrations in pregnant women were associated with increased risks for preterm birth and low birth weight (LBW) newborns [25,26], as well as elevated BMI in children [27]. Vitamin D binding protein (VDBP), previously known as a transport protein for vitamin D and as a regulator of vitamin D metabolism [28], has recently been shown to mediate inflammation and macrophage activation [29]. Maternal vitamin D status was reported to have an impact on birth weight and offspring immunity [30,31].

**Fig 1:**
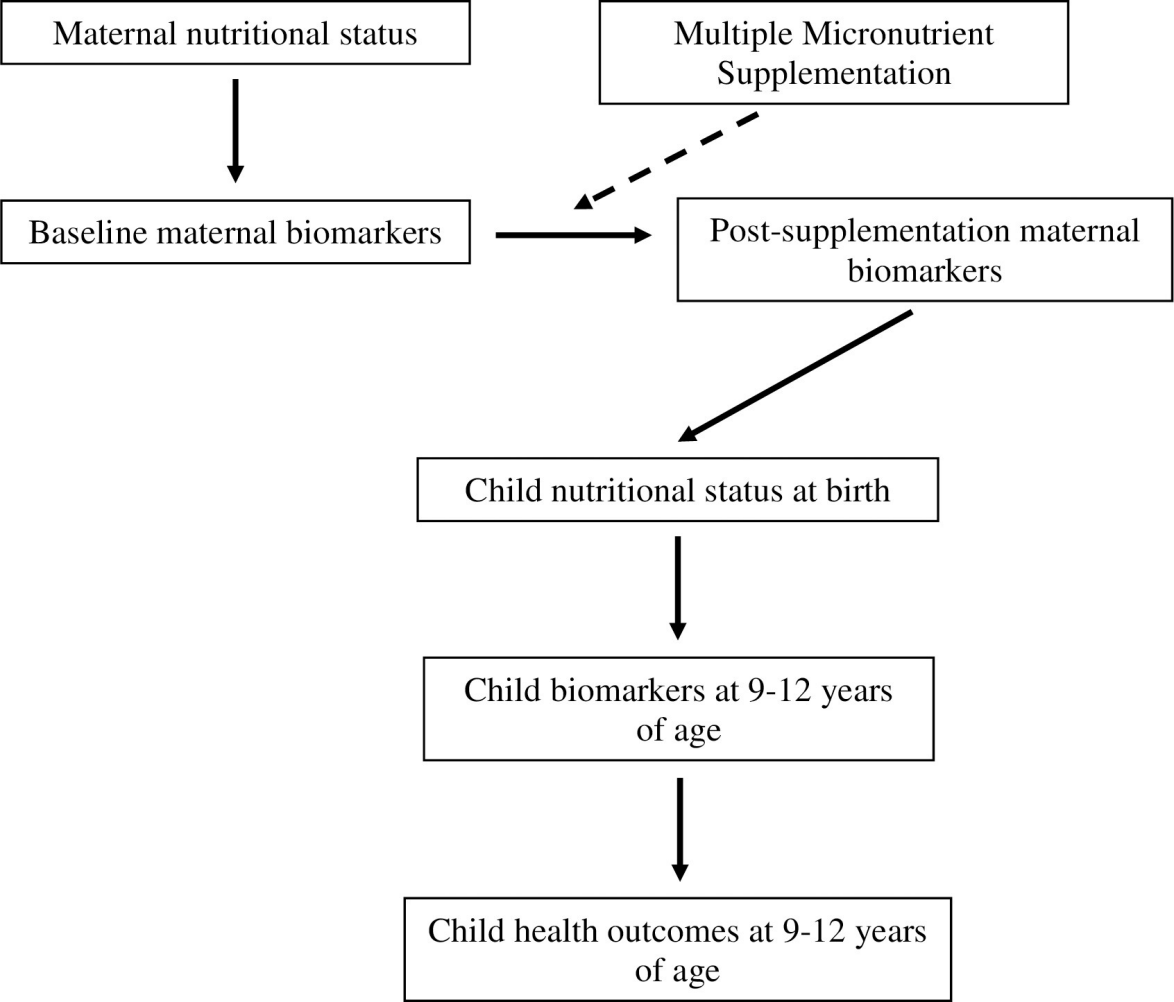
Conceptual framework.

Multiple dietary factors, including micronutrients, have been reported to modulate leptin, adiponectin, RBP4, CRP, and VDBP concentrations [32–37]. Maternal expression patterns for these biomarkers may be associated with expression patterns in their children. To examine these relationships, we studied mother-child dyads from the Supplementation with Multiple Micronutrients Intervention Trial (SUMMIT) in Lombok, Indonesia wherein blood specimens and the relevant data were available from pregnancy as well as their children 9–12 years after birth. The SUMMIT, a randomized trial comparing maternal multiple micronutrients (MMN) supplementation to iron and folic acid (IFA), showed that maternal MMN reduced early infant mortality and LBW [38]. The study also identified multiple risk factors for poor fetal development [39]. A follow-up study of children at 9–12 years of age indicated long term effects of MMN on child cognitive development. We hypothesized that in this cohort: 1. Maternal nutritional status is associated with maternal biomarkers; 2. Maternal MMN supplementation influenced maternal biomarkers; 3. Maternal biomarkers are associated with child biomarkers; 4. Child biomarkers are associated with child health outcomes (Fig 1).

## Materials and methods

### Data collection

The SUMMIT (ISRCTN34151616) was approved by the National Institute of Health Research and Development of the Ministry of Health of Indonesia, the Provincial Planning Department of Nusa Tenggara Barat Province, and the Johns Hopkins Joint Committee on Clinical Investigation, Baltimore, USA; the ten-year follow-up study was approved by the University of Mataram Ethical Research Committee as a certified Institutional Review Board of the National Institute of Health Research and Development of the Ministry of Health of Indonesia; the current study of SUMMIT archived materials was also approved by the Eijkman Institute Research Ethics Commission. Plasma specimens from pregnant women were collected at enrolment before supplementation (baseline) and follow-up specimens at one of four subsequent time points: one month after enrolment, 36 weeks of gestation, one week postpartum, and 12 weeks postpartum (post-supplementation) [40]. Maternal nutritional status was measured at enrollment by mid-upper arm circumference (MUAC), maternal height and maternal hemoglobin (Hb). Child status at age 9–12 years was characterized by height and weight which were converted to BMI-for-age z-score (BMIZ) following World Health Organization norms [41], and by systolic blood pressure (SBP) and diastolic blood pressure (DBP).

### Sample selection

**Fig 2:**
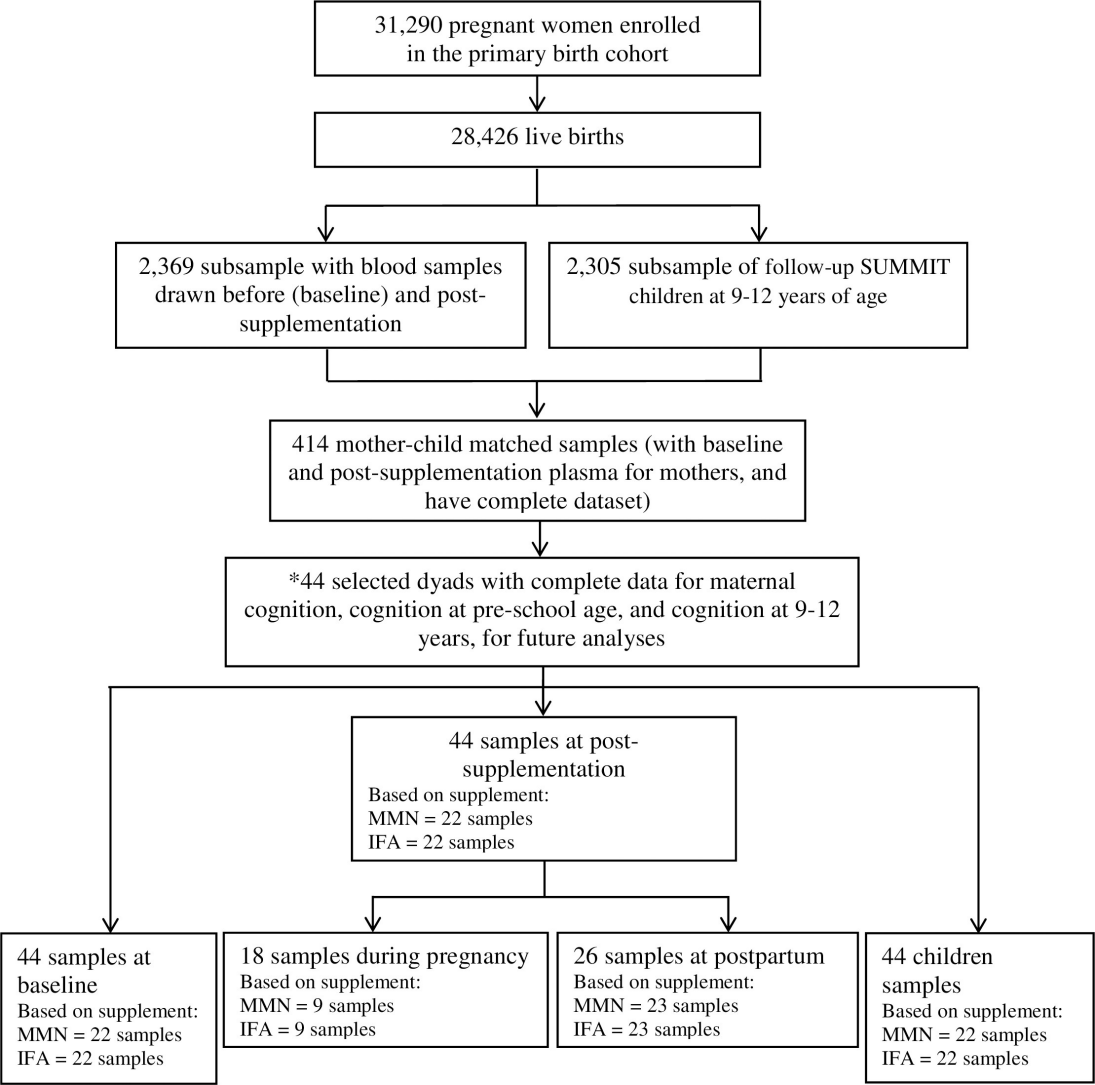
Participant and specimen selectionflow chart. IFA = iron folic acid; MMN = multiple micronutrients. *44 paired maternal-child plasma specimens were selected, consisting of 22 each of the MMN and the IFA groups, with data for maternal cognition, cognition at pre-school age, and cognition at 9–12 years [40].

We selected 414 mother-child dyads from the SUMMIT with plasma samples from three time points: maternal pre-supplementation, maternal post-supplementation, and the child at age 9–12 years. From these, we further selected 44 dyads, consisting of 22 each of the MMN and the IFA groups, who had participated in the studies on maternal cognition [40], cognition at pre-school age [40], and cognition at 9–12 years [42]. This was to optimize the spectrum of outcomes over time that could be included in analyses. Within these 44 dyads, maternal plasma consisted of baseline pre-supplementation samples paired with post-supplementation samples. The post-supplementation samples were collected during pregnancy (either four weeks after enrolment or at 36 weeks gestational age) or postpartum (either one week or 12 weeks postpartum). The post-supplementation during pregnancy group consists of 18 samples (9 from MMN and 9 from IFA groups) and the post-supplementation postpartum group consists of 23 samples (13 from MMN and 13 from IFA groups). A total of 132 maternal and child plasma specimens were analyzed for VDBP, adiponectin, RBP4, CRP, and leptin (Fig 2).

### Multiplex immunoassay

Quantification of leptin, adiponectin, RBP4, CRP, and VDBP was conducted using Luminex^®^ Magnetic Screening Assays (Catalogue number LXSAHM-8, R&D System, Minneapolis, MN, USA) following the manufacturer’s instructions. Plasma samples were diluted according kit requirements and incubated with antibody-coated microspheres, followed by biotinylated detection antibody, and phycoerythrin-labeled streptavidin. The bead immuno-complexes were read using a MagPix CCD Imager (Luminex, Austin, TX, USA) set to the following parameters: events (beads) = 50, sample size = 50 μl. Biomarker concentrations were calculated based on the average of the median fluorescence intensity (MFI) of each duplicate sample.

### Statistical analysis

Data normality for biomarkers was assessed by the Shapiro Wilk test and QQ plots. Biomarkers concentrations were log-transformed to normalize distributions as needed. Normally distributed variables were presented as the mean (±standard deviation). Non-normally distributed variables were presented as the median (interquartile range). Principal component analysis (PCA) was performed to identify specific components of correlation between the five biomarkers as putative composite biomarkers. A component was retained following cross validation by meeting at least two of three criteria: (1) eigenvalue cutoffs defined by Horn’s parallel analysis [43], (2) being robust to outlier prediction based on the squared residual distance Q and Hotelling T^2^ distance as well as pattern of variance explained, (3) frequency of associations in regression analyses that exceeds what would be expected as assessed by the Fisher Exact test. These criteria yielded two retained components for all PCA conducted. Factor loadings greater than absolute value of 0.40 were used to identify biomarkers that loaded on each component as this threshold would imply the observed variable shares more than 15% of its variance (0.40^2^ = 0.16) with the component [44].

The principal component (PC) scores for retained components were computed for each specimen type (baseline, post-supplementation, and child), then normalized to a mean of 0 and standard deviation of 1 and used as either the independent or dependent variable in regression models. To include post-supplementation PC scores in regression analyses, we merged the normalized scores from samples collected during pregnancy and postpartum. Multiple linear regression was used to determine the association of the following variables: maternal PC scores at baseline with maternal nutritional status (association 1), maternal PC scores at baseline with post-supplementation (association 2), maternal PC scores at each time point with child PC scores (association 3), and PC scores of each group with child health outcomes (association 4). Analyses for association 1 were a regression model with maternal PCs at baseline as the dependent variable and baseline maternal hemoglobin, maternal height, maternal mid-upper arm circumference (MUAC), and gestational age at enrolment as independent variables. Association 2 modeled maternal PCs at post-supplementation as the dependent variable and baseline maternal PCs, maternal hemoglobin, maternal height, maternal mid-upper arm circumference (MUAC), and type of supplement (MMN or IFA) as the independent variables. We analyzed the interaction of MMN supplementation with maternal PCs at baseline and maternal PCs at post-supplementation. In the regression model for association 3, the dependent variables were child PCs, while the independent variables were maternal PCs at baseline and post-supplementation, and baseline maternal hemoglobin, maternal height, maternal MUAC, birth weight, child gender (boy or girl), and type of supplement (MMN or IFA). Association 4 modeled maternal and child PCs, baseline maternal hemoglobin, maternal height, maternal MUAC, birth weight, child gender (boy or girl), and type of supplement (MMN or IFA) as the independent variables when the BMIZ was the dependent variable, with additional adjustment for child BMIZ when the systolic blood pressure (SBP) and diastolic blood pressure (DBP) were the dependent variables. All regression analyses were performed using R-Project for Statistical Computing version 3.4.0 and SAS 9.4. A *p*-value of less than 0.05 was considered significant.

## Results

### Baseline characteristics of subjects

The baseline characteristics of mother-child dyads were collected during the SUMMIT and its follow up studies, as shown in Table 1. Pregnant women who received MMN supplementation had similar characteristics to those receiving IFA. The characteristics of the children at 9–12 years of age whose mothers received MMN or IFA supplementation were also similar to the overall SUMMIT enrollees, as were the general characteristics of women in this study [38,45].

**Table 1:** Baseline characteristics of mother-child dyads.

Characteristics	MMN (N = 22)	IFA (N = 22)	*p*-value
**Mothers**			
Age (years) ^¶^	25.0 (20.0–26.5)	25.5 (20.5–30.0)	0.251
Parity (number of births) ^‡^			
0	8 (36)	5 (23)	0.509
≥ 1	14 (64)	17 (77)	
Height (cm) ^¶^	151.4 (149.3–153.6)	149.8 (148.7–152.6)	0.231
Mid-upper arm circumference (mm) ^¶^	239.5 (228.2–253.0)	245.0 (230.2–253.1)	0.503
Haemoglobin at enrolment (g/dL) ^¶^	11.1 (10.3–12.0)	11.3 (10.4–11.9)	0.842
Gestational age at enrolment (weeks) ^¶^	16.5 (9.5–24.1)	14.6 (12.3–18.7)	0.734
**Children**			
Gender (M/F)	13/9	10/12	0.546
BMI-for-age z-scores ^†^	−0.7x (±1.0x)	−0.8x (±1.1x)	0.678
Systolic blood pressure (mmHg) ^†^	110.0 (±11.3)	104.4 (±7.8)	0.525
Diastolic blood pressure (mmHg) ^†^	65.0 (±9.8)	63.4 (±5.3)	0.067
Birth weight (g) ^¶^	3300 (2925–3500)	3000 (2825–3450)	0.350
Gestational age at birth (weeks) ^¶^	39.1 (36.9–40.1)	39.6 (38.1–40.9)	0.231

^¶^: median (interquartile range).

^†^: mean (±standard deviation).

^‡^: n (percentage). MMN: multiple micronutrients supplement. IFA: iron and folic acid supplement.

### Biomarker concentrations of women and children

The median values of the selected biomarkers are summarized in Table 2. The biomarker concentrations for each supplement are presented in S1 Table.

**Table 2:** Biomarker concentrations of women during baseline, post-supplementation during pregnancy, post-supplementation at postpartum, and in children.

Biomarker	Baseline (N = 44)	Post-supplementation during pregnancy (N = 18)	Post-supplementation at postpartum (N = 26)	Children (N = 44)
VDBP (μg/mL)	52.8 (32.6–86.0)	34.1 (21.3–49.0)	39.5 (29.4–102.4)	19.1 (15.9–24.7)
Adiponectin (μg/mL)	3.0 (2.0–4.1)	2.5 (2.1–2.9)	3.3 (2.3–4.3)	5.2 (4.6–6.5)
RBP4 (μg/mL)	27.3 (22.1–35.9)	20.3 (16.6–32.3)	39.4 (28.8–47.1)	24.2 (19.6–28.9)
CRP (μg/mL)	2.0 (0.6–3.4)	1.3 (0.4–2.2)	0.5 (0.1–1.2)	0.2 (0.1–0.6)
Leptin (ng/mL)	8.2 (4.8–13.8)	15.0 (10.5–21.4)	3.5 (2.1–5.7)	3.1 (2.4–5.8)

VDBP: vitamin D binding protein. RBP4: retinol binding protein. CRP: C-reactive protein. Data in median (interquartile range).

### Principal Component Analysis (PCA) to identify composite biomarker components

Table 3 shows the results of principal component analysis. The first two PCs were retained for further analyses based on the criteria detailed in Materials and Methods. For maternal PCA, the first two PCs explained 60% (PC1 = 39.5%, PC2 = 20.5%), 77.6% (PC1 = 52.1%, PC2 = 25.5%), and 60.5% (PC1 = 36.9%, PC2 = 23.6%) of the total variance for baseline, post-supplementation during pregnancy and post-supplementation postpartum groups, respectively. For child PCA, the first two PCs explained 63.2% (PC1 = 40.0%, PC2 = 23.2%). Each group had distinctive component patterns based on biomarker loadings. For the maternal baseline pregnancy (bp) group, PC1 consisted of negative loadings for VDBP (D), adiponectin (A), and RBP4 (R) (bp.pc1.D↓A↓R↓), while PC2 consisted of negative loadings for CRP (C) and positive for leptin (L) (bp.pc2.C↓L↑). The PC1 for post-supplementation during pregnancy (dp) was comprised of positive loadings for VDBP, adiponectin, and RBP4 (dp.pc1.D↑A↑R↑), while PC2 was comprised of positive loadings for adiponectin and leptin (dp.pc2.A↑L↑). For the post-supplementation postpartum group (pp), PC1 was characterized by negative loadings for VDBP, RBP4, and leptin (pp.pc1.D↓R↓L↓), and PC2 by positive loadings for adiponectin, CRP and leptin (pp.pc2.A↑C↑L↑). The child (ch) PC1 consisted of positive loadings for VDBP, RBP4 and CRP (ch.pc1.D↑R↑C↑), while the PC2 consisted of negative loadings for VDBP, and positive for adiponectin and leptin (ch.pc2.D↓A↑L↑). The complete principal component analysis results of maternal biomarkers and child biomarkers are presented in S2–S5 Tables.

**Table 3:** Principal component analysis of biomarkers for maternal baseline, maternal follow-up, and for children.

	Baseline (N = 44)		Post-supplementation during pregnancy (N = 18)		Post-supplementation at postpartum (N = 26)		Children (N = 44)	
	PC1	PC2	PC1	PC2	PC1	PC2	PC1	PC2
Eigenvalues	1.974	1.026	2.607	1.277	1.846	1.181	1.997	1.163
% variance accounted for	39.484	20.518	52.134	25.540	36.927	23.620	39.950	23.268
Loadings								
Log VDBP	**−0.407**	0.056	**0.586**	**−**0.057	**−0.585**	0.170	**0.464**	**−0.529**
Log Adiponectin	**−0.569**	**−**0.222	**0.427**	**0.533**	0.310	**0.536**	0.157	**0.609**
Log RBP4	**−0.519**	0.368	**0.496**	0.303	**−0.609**	**−**0.077	**0.600**	0.086
Log CRP	**−**0.390	**−0.679**	0.389	**−**0.397	0.111	**0.689**	**0.497**	**−**0.226
Log Leptin	**−**0.299	**0.592**	**−**0.280	**0.680**	**−0.422**	**0.452**	0.391	**0.540**

PC: principal component. VDBP: vitamin D binding protein. RBP4: retinol binding protein. CRP: C-reactive protein. Principal component analysis (PCA) was performed to identify composite biomarker components. Components were retained based on criteria described in Materials and Methods. Loadings >0.40, in bold, were used to define and characterize the component [44].

### Associations of maternal baseline nutrition characteristics with maternal baseline pregnancy components

Linear regression analyses between maternal PCs at baseline and maternal nutrition status showed that PC1 bp.pc1.D↓A↓R↓ had a mild negative association with reduced MUAC in both unadjusted (β = −0.017, *p* = 0.036) and adjusted (β = −0.020, *p* = 0.025) models. Meanwhile, PC2 bp.pc2.C↓L↑ displayed a mild positive association with increased MUAC in unadjusted analysis (β = 0.013, *p* = 0.023), and tendency, though not significant, for association in adjusted analysis (β = 0.012, *p* = 0.068) (Table 4). Regression analyses between individual maternal biomarkers and maternal nutritional status are presented in S6 Table.

**Table 4:** Associations of maternal baseline nutrition characteristics with maternal baseline pregnancy components.

	bp.pc1.D↓A↓R↓ (n = 44)				bp.pc2.C↓L↑ (n = 44)			
	Unadjusted		Adjusted		Unadjusted		Adjusted	
	B	*p*	B	*p*	B	*p*	B	*p*
Hb at baseline	0.005	0.975	0.036	0.835	0.162	0.148	0.065	0.617
Height (cm)	-0.075	0.263	-0.053	0.400	-0.008	0.865	-0.016	0.732
MUAC (mm)	-0.017	**0.036**	-0.02	**0.025**	0.013	**0.023**	0.012	0.068
Gestational age (weeks)	-0.043	0.146	-0.052	0.095	-0.014	0.499	-0.001	0.964

PC: principal component; bp.pc1.D↓A↓R↓: baseline maternal PC1; bp.pc2.C↓L↑: baseline maternal PC2; D: vitamin D binding protein; A: adiponectin; R: retinol binding protein 4; C: C-reactive protein; L: leptin; ↓: decrease; ↑: increase; B: coefficient of regression; Hb: hemoglobin; MUAC: mid-upper arm circumference. Association analyses were performed using unadjusted and adjusted linear models. For adjusted regressions, the dependent variables were baseline maternal PCs and the independent variables were maternal Hb at baseline, maternal height, maternal MUAC at baseline, and gestational age at enrolment. Significant *p* values <0.05 are in bold.

### Associations of maternal baseline pregnancy components, maternal nutrition, and multiple micronutrient supplementation with post-supplementation components

**Fig 3:**
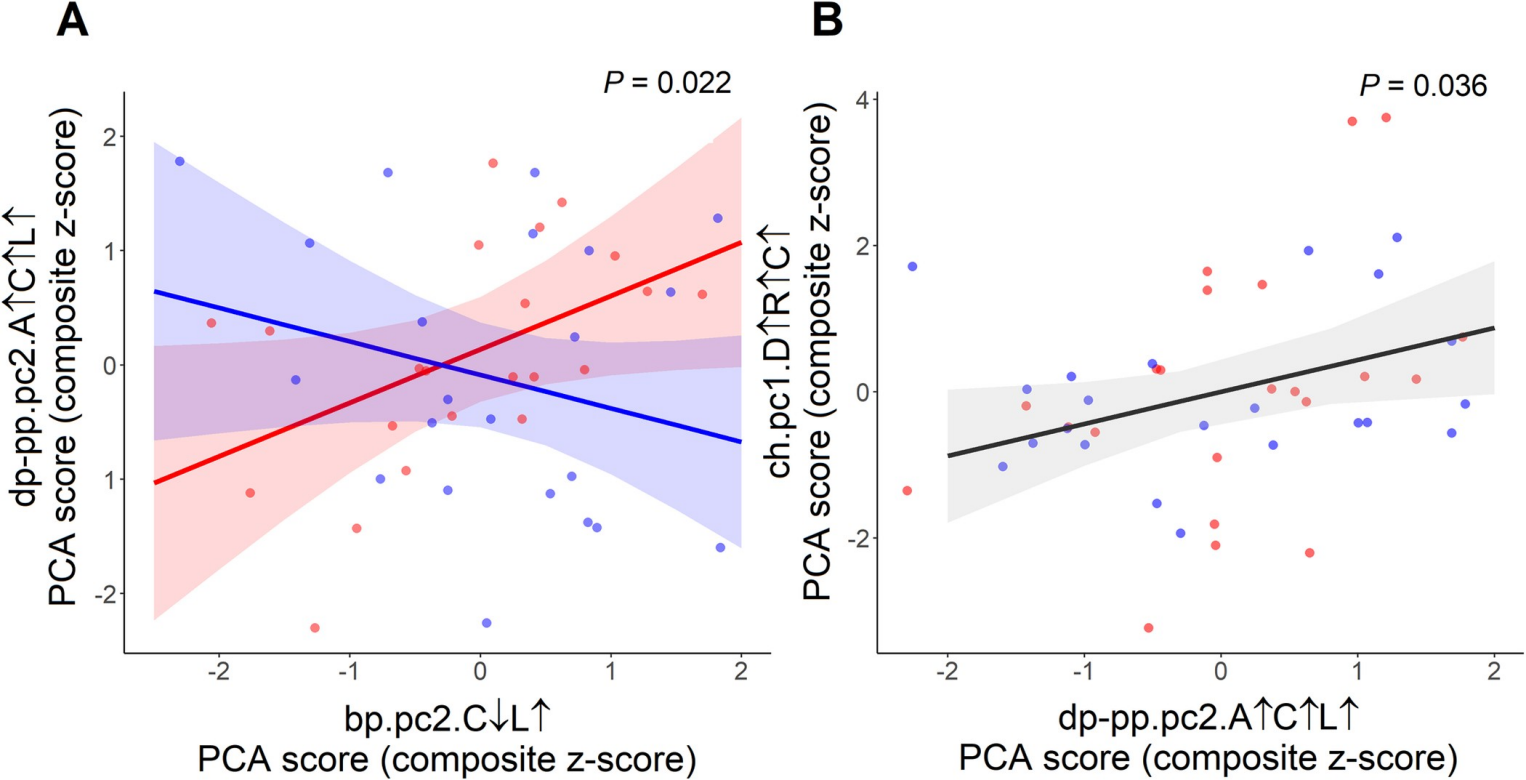
A. Maternal multiple micronutrient supplementation affects associations between maternal biomarker components. Interaction between baseline maternal PC2 bp.pc2.C↓L↑ and supplementation type with post-supplementation maternal PC2 dp-pp.pc2.A↑C↑L↑. B. Effect of maternal biomarker component on child biomarker component. Association of maternal PC2 dp-pp.pc2.A↑C↑L↑ and child PC1 ch.pc1.D↑R↑C↑. Blue line and blue dots: MMN supplementation; Red line and red dots: IFA supplementation.

Regression analyses for the associations between maternal PCs at baseline and at post-supplementation are presented in Table 5. Baseline maternal PC1 bp.pc1.D↓A↓R↓ was negatively associated with the post-supplementation maternal PC2 dp-pp.pc2.A↑C↑L↑ (β = −0.315, *p* = 0.028). A negative association was also found between the baseline maternal PC2 bp.pc2.C↓L↑ and the post-supplementation maternal PC1 dp-pp.pc1.D↑↓A↑R↑↓L↓ (β = −0.518, *p* = 0.022). Of particular interest were analyses incorporating an interaction term between each PC and supplementation type (IFA or MMN), which revealed that MMN caused baseline bp.pc2.C↓L↑ to be negatively associated with post-supplementation maternal PC2 dp-pp.pc2.A↑C↑L↑, whereas these components were positively associated for the IFA group (*p* interaction = 0.022) (Fig 3A), Analysis of maternal baseline and post-supplementation biomarkers is shown in S7 Table.

**Table 5:** Associations of maternal baseline pregnancy components, maternal nutrition, and multiple micronutrient supplementation with and post-supplementation components.

	dp-pp.pc1.D↑↓A↑R↑↓L↓ (n = 44)				dp-pp.pc2.A↑C↑L↑ (n = 44)			
	Unadjusted		Adjusted		Unadjusted		Adjusted	
	B	*p*	B	*p*	B	*p*	B	*p*
bp.pc1.D↓A↓R↓	-0.269	0.088	-0.29	0.083	-0.284	**0.015**	-0.315	**0.028**
bp.pc2.C↓L↑	-0.516	**0.016**	-0.518	**0.022**	0.084	0.616	0.066	0.719
Hb at baseline (g/dL)	-0.241	0.132	-0.132	0.421	0.062	0.61	0.042	0.762
Height (cm)	-0.07	0.312	-0.111	0.100	-0.015	0.772	-0.026	0.646
MUAC (mm)	-0.011	0.204	-0.003	0.731	0.009	0.166	0.001	0.889
MMN supplementation	0.648	0.14	0.723	0.100	-0.121	0.718	-0.279	0.445
Interaction model:								
bp.pc1.D↓A↓R↓*MMN	-0.281	0.376	**−**0.257	0.395	-0.121	0.604	**−**0.149	0.531
bp.pc2.C↓L↑*MMN	0.240	0.558	0.315	0.438	-0.799	**0.016**	**−**0.761	**0.022**

PC: principal component; bp.pc1.D↓A↓R↓: baseline maternal PC1; bp.pc2.C↓L↑: baseline maternal PC2; dp-pp.pc1.D↑↓A↑R↑↓L↓: post-supplementation maternal PC1; dp-pp.pc2.A↑C↑L↑: post-supplementation maternal PC2; D: vitamin D binding protein; A: adiponectin; R: retinol binding protein 4; C: C-reactive protein; L: leptin; ↓: decrease; ↑: increase; ↑↓: increased post-supplementation during pregnancy and decreased post-supplementation at postpartum; B: coefficient of regression; Hb: hemoglobin; MUAC: mid-upper arm circumference; MMN: multiple micronutrients. Analysis were performed using unadjusted and adjusted linear models. For adjusted regressions, the dependent variables were post-supplementation maternal PCs, and the independent variables were baseline maternal PCs, maternal Hb at baseline, maternal height, maternal MUAC at baseline, and MMN/IFA supplementation. For interaction (*) we added the terms baseline maternal PC1*MMN/IFA supplementation and baseline maternal PC2*MMN/IFA supplementation. Significant *p* values <0.05 are in bold.

### Associations of maternal components and child characteristics with child biomarker components

We found that post-supplementation maternal PC2 dp-pp.pc2.A↑C↑L↑ was positively associated with child PC1 ch.pc1.D↑R↑C↑ (β = 0.439, *p* = 0.036) (Fig 3B). As shown in Table 6, the child PC1 ch.pc1.D↑R↑C↑ was also negatively associated with birth weight (β = −0.826, *p* = 0.036). The child PC2 ch.pc2.D↓A↑L↑ showed a mild negative association with maternal height (β = −0.097, *p* = 0.030), and strong negative association with male gender (β = −0.958, *p* = 0.002) (Table 6). The association of individual child biomarkers with maternal biomarkers at baseline and post-supplementation are shown in S8 Table and S9 Table.

**Table 6:** Association of maternal components and child characteristics with child biomarker components.

	ch.pc1.D↑R↑C↑ (n = 44)				ch.pc2.D↓A↑L↑ (n = 44)			
	Unadjusted		Adjusted		Unadjusted		Adjusted	
	B	*p*	B	*p*	B	*p*	B	*p*
bp.pc1.D↓A↓R↓	-0.094	0.546	0.243	0.195	0.041	0.732	-0.010	0.932
bp.pc2.C↓L↑	0.303	0.156	0.292	0.237	0.230	0.160	-0.043	0.774
dp-pp.pc1.D↑↓A↑R↑↓L↓	0.011	0.939	0.204	0.242	-0.040	0.727	-0.103	0.330
dp-pp.pc2.A↑C↑L↑	0.392	**0.046**	0.439	**0.036**	0.189	0.214	0.168	0.182
Hb at baseline (g/dL)	0.220	0.158	0.015	0.932	-0.124	0.301	-0.072	0.511
Height (cm)	0.066	0.324	0.090	0.204	-0.125	**0.012**	-0.097	**0.030**
MUAC (mm)	0.018	**0.023**	0.018	0.091	0.005	0.441	0.001	0.925
Birth weight (kg)	-0.685	0.074	-0.826	**0.036**	0.203	0.496	0.347	0.142
Gender: Boy	-0.035	0.936	0.496	0.299	-0.566	0.082	-0.958	**0.002**
MMN supplementation	-0.073	0.866	-0.092	0.841	0.006	0.986	0.328	0.249

PC: principal component; bp.pc1.D↓A↓R↓: baseline maternal PC1; bp.pc2.C↓L↑: baseline maternal PC2; dp-pp.pc1.D↑↓A↑R↑↓L↓: post-supplementation maternal PC1; dp-pp.pc2.A↑C↑L↑: post-supplementation maternal PC2; ch.pc1.D↑R↑C↑: child PC1; ch.pc2.D↓A↑L↑: child PC2; D: vitamin D binding protein; A: adiponectin; R: retinol binding protein 4; C: C-reactive protein; L: leptin; ↓: decrease; ↑: increase; ↑↓: increased post-supplementation during pregnancy and decreased post-supplementation at postpartum; B: coefficient of regression; Hb: hemoglobin; MUAC: mid-upper arm circumference; MMN: multiple micronutrients. Analysis was performed using unadjusted and adjusted linear models. For adjusted regressions, the dependent variables were child PCs, and the independent variables were baseline maternal PCs, post-supplementation maternal PCs, maternal Hb at baseline, maternal height, maternal MUAC at baseline, birth weight, child's gender (boy/girl), and MMN/IFA supplementation. Significant *p* values <0.05 are shown in bold.

### Association of child health outcomes with maternal and child biomarker components

**Fig 4:**
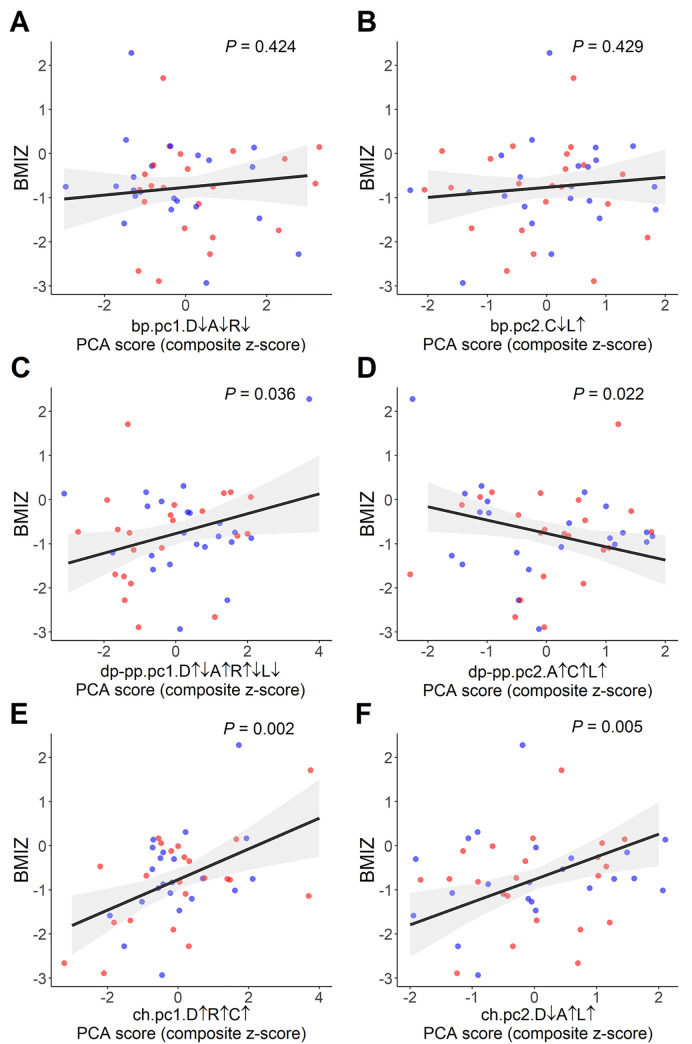
Association of maternal and child biomarker components with child BMIZ. **A-B**. Maternal baseline PC1 bp.pc1.D↓A↓R↓ and PC2 bp.pc2.C↓L↑. **C-D**. Maternal PC1 dp-pp.pc1.D↑↓A↑R↑↓L↓ and PC2 dp-pp.pc2. A↑C↑L↑. **E-F**. Child PC1 ch.pc1.D↑R↑C↑ and PC2 ch.pc2.D↓A↑L↑. Blue dots: MMN supplementation; Red dots: IFA supplementation.

We then analyzed the association of maternal and child biomarker PC scores with child health outcomes (BMIZ, SBP, and DBP) as seen in Table 7. We found that child BMIZ was negatively associated with the maternal dp-pp.pc2.A↑C↑L↑ (β = –0.302, *p =* 0.022), and positively associated with maternal pp.pc1.D↑↓A↑R↑↓L↓ (β = 0.224, *p =* 0.036), ch.pc1.D↑R↑C↑ (β = 0.347, *p* = 0.002), and ch.pc2.D↓A↑L↑ (β = 0.515, *p* = 0.005) (Fig 4). With respect to maternal characteristics, we observed that child BMIZ was negatively associated with baseline maternal Hb (β = –0.280, *p* = 0.010), and mildly positively associated with maternal MUAC (β = 0.014, *p* = 0.027). No significant associations were found with child SBP and DBP. The association of child health outcome with maternal biomarkers and child biomarkers are shown in S10 Table (maternal biomarkers at baseline) and S11 Table (maternal biomarkers at post-supplementation) and S12 Table (child biomarkers).

**Table 7:** Associations of maternal and child components and nutritional characteristics with child body mass index and blood pressure.

	Child's outcome											
	BMIZ (n = 44)				SBP (n = 43)				DBP (n = 43)			
	Unadjusted		Adjusted		Unadjusted		Adjusted		Unadjusted		Adjusted	
	B	*p*	B	*p*	B	*p*	B	*p*	B	*P*	B	*p*
bp.pc1.D↓A↓R↓	-0.063	0.581	0.088	0.424	0.564	0.609	1.100	0.445	0.486	0.571	0.853	0.438
bp.pc2.C↓L↑	0.081	0.610	0.114	0.429	1.272	0.415	1.050	0.606	0.186	0.879	-0.427	0.783
dp-pp.pc1.D↑↓A↑R↑↓L↓	0.144	0.191	0.224	**0.036**	1.352	0.201	1.968	0.185	1.222	0.136	1.369	0.227
dp-pp.pc2.A↑C↑L↑	-0.067	0.649	-0.302	**0.022**	-1.954	0.164	-2.788	0.126	-0.432	0.695	-0.605	0.658
ch.pc1.D↑R↑C↑	0.368	**0.001**	0.347	**0.002**	1.991	0.064	2.123	0.199	1.696	**0.042**	0.894	0.474
ch.pc2.D↓A↑L↑	0.163	0.269	0.515	**0.005**	1.113	0.441	2.097	0.428	2.289	**0.037**	2.403	0.237
Hb at baseline (g/dL)	-0.155	0.176	-0.280	**0.010**	0.273	0.807	0.594	0.692	-0.042	0.962	1.073	0.352
Height (cm)	0.061	0.210	0.063	0.165	0.314	0.509	0.328	0.592	0.042	0.909	0.402	0.392
MUAC (mm)	0.009	0.125	0.014	**0.026**	0.030	0.612	0.018	0.842	0.027	0.558	-0.013	0.851
Birth weight (kg)	0.000	0.540	-0.046	0.852	0.476	0.864	2.667	0.415	-2.179	0.309	-0.912	0.714
Gender: Boy	-0.277	0.381	0.540	0.104	-1.077	0.728	-0.715	0.875	-2.988	0.211	-2.332	0.503
MMN supplementation	0.132	0.678	-0.080	0.766	5.637	0.063	3.684	0.322	1.592	0.508	0.587	0.835
Child BMIZ					4.064	**0.007**	1.035	0.670	3.444	**0.003**	1.990	0.288

PC: principal component; bp.pc1.D↓A↓R↓: baseline maternal PC1; bp.pc2.C↓L↑: baseline maternal PC2; dp-pp.pc1.D↑↓A↑R↑↓L↓: post-supplementation maternal PC1; dp-pp.pc2.A↑C↑L↑: post-supplementation maternal PC2; ch.pc1.D↑R↑C↑: child PC1; ch.pc2.D↓A↑L↑: child PC2; D: vitamin D binding protein; A: adiponectin; R: retinol binding protein 4; C: C-reactive protein; L: leptin; ↓: decrease; ↑: increase; ↑↓: increased post-supplementation during pregnancy and decreased post-supplementation at postpartum; B: coefficient of regression; Hb: hemoglobin; MUAC: mid-upper arm circumference; MMN: multiple micronutrients; BMI: body mass index; SBP: systolic blood pressure; DBP: diastolic blood pressure. Analysis was performed using unadjusted and adjusted linear models. For adjusted regressions, the dependent variables were BMIZ, SBP, DBP, and the independent variables were baseline maternal PCs, post-supplementation maternal PCs, child PCs, maternal Hb at baseline, maternal height, maternal MUAC at baseline, birth weight, child's gender (boy/girl), MMN/IFA supplementation, and child BMIZ for models with SBP and DBP as dependent variables. Significant *p* values <0.05 are indicated in bold.

## Discussion

To our knowledge, few studies have explored the association of maternal metabolic biomarkers during pregnancy and postpartum with child metabolic biomarkers at age 9–12 years. Moreover, because biomarkers may not work independently, but in concert, potential interactions between composite biomarker components and outcomes may better represent the complexity of their effects. We therefore utilized PCA to construct composite components of biomarkers that represented their covariance structure and analyzed the associations of the resulting components and other characteristics, with downstream components and health indicators.

PCA showed that maternal biomarkers at baseline and post-supplementation during pregnancy and postpartum had distinctive component structures, indicating that gestational age may influence the maternal biomarker patterns. We found that increased maternal MUAC was associated with lower baseline maternal PC1 bp.pc1.D↓A↓R↓. This is consistent with previous reports where nutritional status measured by BMI was positively correlated with leptin, adiponectin, and RBP4 concentrations [46–48], though these studies were not done in pregnant women.

We also found that maternal biomarker PCs at baseline were associated with biomarker PCs at post-supplementation, although associations at these timepoints between individual biomarkers were observed only for adiponectin and RBP4 (S8 Table). This suggests that biomarkers may indeed have stronger influence working in concert as components in a networked biological system. In this context it is intriguing that maternal MMN supplementation interacted with maternal baseline PC bp.pc2.C↓L↑ to strongly decrease post-supplementation PC dp-pp.pc2.A↑C↑L↑. This is consistent with reports of vitamin C and E supplementation reducing CRP concentrations [36,49], and vitamin D supplementation reducing serum leptin [50].

We observed that maternal PC dp-pp.pc2.A↑C↑L↑ was associated with higher child PC ch.pc1.D↑R↑C↑ at 9–12 years of age and with lower child BMIZ. This suggests co-elevation of adiponectin, CRP, and leptin in pregnancy may lead to co-elevation of VDBP, RBP4, and CRP in the child. Moreover, maternal MMN might therefore tend to decrease VDBP, RBP4, and CRP in the child, which could favor lower BMIZ, as we observed in Table 7, and possibly leaner growth. However, we note that a decrease in PC dp-pp.pc2.A↑C↑L↑ as shown in Table 7 might also favour higher BMIZ.

Previous studies showed that maternal leptin concentration was correlated with child leptin concentration in cord blood [23,51] and serum of 9-years old children [52]. Postpartum maternal biomarkers may be associated with child biomarkers through breast milk, in agreement with a previous study that reported a correlation between leptin concentration in breast milk with its concentration in maternal serum and infant weight gain [53]. Although genetics was also reported to have moderate influence on variation of biomarkers concentration [54,55], environmental factors such as nutrition, including micronutrients, and infection have been reported to more strongly modulate adipocytokines and inflammatory markers [32–37,56]. Our analysis did not include the influence of dietary intake on biomarkers concentrations, which could reveal additional associations. Daily nutrient-dense food intake should remain the principal source of micronutrients. In this study, we did not include analysis of dietary intake, and further analyses of SUMMIT dietary data in this context may yield additional insights.

BMI-for-age z-score represents nutritional and health conditions in children and adolescents [57]. Our study showed that maternal and child biomarker PCs were associated with child BMIZ. This is in line with previous studies that reported BMIZ in children was correlated with biomarkers concentrations, such as leptin [58] and RBP4 concentrations [59]. In our study, the average BMIZ was below the WHO standard for a healthy population [41], which means the children tended to be underweight. However, BMIZ is a modifiable factor which can be improved by nutritional and behavioral interventions [60]. Thus, maternal MMN supplementation during pregnancy might indirectly influence child BMIZ considering that our results indicated that MMN modified the association between maternal baseline and maternal post-supplementation biomarker PC scores, while maternal post-supplementation PC scores were associated with child biomarker PC scores and BMIZ.

It has been suggested that pre-pregnancy and pregnancy nutritional status have long term effects on health outcomes of children. Both maternal height and MUAC were positively associated with child PC scores, although these were not significant. Maternal Hb during pregnancy and height were also associated with child BMIZ. These results support the potential influence of maternal nutritional status on long term child metabolism and health. This notion has been previously reported wherein maternal BMI was correlated with child BMI [61] and weight for height z-score (WHZ) [62]. Maternal BMI was also reported to be associated with infant serum leptin values [48]. Therefore, our findings also highlight the importance of optimal macronutrient intake during pregnancy that would improve maternal nutritional status and child health later in life [63]. In this context, the reported greater impact of maternal MMN on birth weight in well-nourished women is noteworthy [38].

We proposed that maternal biomarkers of adipocytokines and inflammatory markers could influence the same biomarkers in the child through the interactions of immunologic and metabolic factors. Adiponectin, RBP4, CRP, and leptin play important roles in regulating metabolism, energy homeostasis, and inflammatory responses, while VDBP has a role in modulating immune and inflammatory response. The immune and metabolic system have co-evolved to signal each other and form complex networks in response to environmental exposures, such as the secretion of leptin and adiponectin that are contra-regulated [64,65]. Transfer of immune and metabolic properties between mother and child occurs through the placenta [23,66], and through breast milk during the neonatal period [53]. Together, these immune-metabolic signals provide innate and adaptive immunity, and influencing the metabolic homeostasis of the newborn. The transmission of these cross-generational immune and metabolic properties may be modified via optimal macronutrient and micronutrient intake during pregnancy and postpartum. Maternal adverse conditions, such as malnutrition or infection may modify these signals and alter newborn immunity, consequently influencing newborn and infant health, and possibly later life [67,68].

It is remarkable that despite the relatively small set of specimens analyzed in this study, significant and interpretable associations were observed, suggesting that the biomarker components exhibit strong influence. We also note that the overall associations identified through components tended, although not always, to be more frequent and stronger than for individual biomarkers alone. Replication of this study’s findings would be warranted. In addition, due to the multiple hypotheses tested, the multiple comparisons in the study were unavoidable, but again we note the frequency of associations exceeds that which would be expected by chance as assessed by the Fisher Exact test on PCs not retained for analyses which would represent random data. To our knowledge, this is the first study suggesting an effect of maternal MMN supplementation on the child outcomes via modulation of the mother’s biomarkers. We suggest that specific effects of a particular micronutrient or of MMN overall cannot be determined based on a single biomarker, as there would be many pathways involved. Therefore, analyzing the effect of a composite biomarker component may be more relevant, as conducted here.

While the above findings suggest associations between maternal and child biomarker status as well as a role of MMN in this relationship, there are several limitations of the study. First, the limited sample size yielded limited statistical power, precluding more detailed analyses. For example, we could not assess the outcome of gestational age at birth. Similarly, in some cases the distribution of predictors in regression models may not have adequately represented the full spectrum of values. The impact of this in many cases was greater variance, thereby limiting associations. In addition, other potentially important covariates were not included, such as dietary intake or recent infections, or blood samples from children at younger ages that could be analyzed. Finally, while we utilized PCA to discern components, this approach would not be able to identify localized clustering of biomarkers in the n-dimensional space. Other techniques such as k-means clustering or uniform manifold approximation and projection (UMAP) may also be useful and would require greater sample size. Nevertheless, the results herein are suggestive, and additional confirmation would be warranted.

In the SUMMIT, MMN supplementation compared to IFA improved birth and health outcomes [38]. The IFA contained 30 mg iron and 400 μg folic acid, and the MMN followed the UNIMMAP formulation that contained 30 mg iron and 400 μg folic acid along with 800 μg retinol, 200 IU vitamin D, 10 mg vitamin E, 70 mg ascorbic acid, 1.4 mg vitamin B1, 1.4 mg vitamin B2, 18 mg niacin, 1.9 mg vitamin B6, 2.6 μg vitamin B12, 15 mg zinc, 2 mg copper, 65 μg selenium, and 150 μg iodine. Deficiencies of these micronutrients have been associated with adverse pregnancy outcomes. For example, vitamin A deficiency may lead to night blindness [69], vitamin D deficiency is associated with preeclampsia, insulin resistance, and gestational diabetes mellitus [70]. Vitamin E and C are antioxidants to prevent pre‐eclampsia [71]. Vitamin B1 deficiency may cause of IUGR [72]. Vitamins B6 and B12 play important roles in maternal health as well as fetal development and physiology [73]. Deficiencies of minerals such as zinc, selenium, copper and iodine have also been associated with complications in pregnancy, childbirth or fetal development [74–76]. We recently showed that increases in mitochondrial DNA copy number during pregnancy are associated with LBW, and that maternal MMN supplementation stabilized mitochondrial DNA copy number in peripheral blood mononuclear cells of SUMMIT women, indicating its effects on improved energy efficiency and reduced oxidative damage [77,78].

In conclusion, the results herein suggest that biomarkers of adipocytokines and inflammatory mediators during pregnancy comprise components that may influence downstream biomarker components in pregnancy and in children 9–12 years later, along with child BMIZ. Moreover, MMN supplementation may affect the relationship between components, and further influence child BMIZ score. Improving maternal nutritional status may improve child health not only at birth, but also during childhood, and into adulthood.

## Supporting information

S1 Checklist STROBE statement—checklist of items that should be included in reports of *cross-sectional studies*. (DOCX)

S1 Fig Screeplot of maternal baseline PCA. (DOCX)

S2 Fig Screeplot of maternal post-supplementation during prengancy PCA. (DOCX)

S3 Fig Screeplot of maternal post-supplementation at post-partum PCA. (DOCX)

S4 Fig Screeplot of children PCA. (DOCX)

S5 Fig Cross validation of cumulative variance. Cross validation was performed using ‘mdatools’ package. Blue line: cumulative variance of PCA result. Red line: cumulative variance of cross validation result.
(DOCX)

S6 Fig Correlation map between principle components and all variables. (DOCX)

S1 Table Biomarker concentrations of pregnant women during baseline, post-supplementation during pregnancy, post-supplementation at post-partum, and in children. (DOCX)

S2 Table Principal component analysis results of maternal biomarkers at baseline. (DOCX)

S3 Table Principal component analysis results of maternal biomarkers post-supplementation during pregnancy. (DOCX)

S4 Table Principal component analysis results of maternal biomarkers post-supplementation at post-partum. (DOCX)

S5 Table Principal component analysis results of children’s biomarkers. (DOCX)

S6 Table Association between maternal biomarkers at baseline and maternal nutritional status. (DOCX)

S7 Table Association between maternal biomarkers at baseline and post-supplementation. (DOCX)

S8 Table Association between child biomarkers and maternal biomarkers at baseline. (DOCX)

S9 Table Association between child biomarkers and maternal biomarkers at post-supplementation. (DOCX)

S10 Table Association between child's outcome and maternal biomarkers at baseline. (DOCX)

S11 Table Association between child's outcome and maternal biomarkers at post-supplementation. (DOCX)

S12 Table Association between child's outcome and child's biomarkers. (DOCX)

S13 Table Spearman correlation of maternal biomarkers at baseline and post-supplementation during pregnancy. (DOCX)

S14 Table Spearman correlation of maternal biomarkers at baseline and post-supplementation at post-partum. (DOCX)
