# Supplementary figures and images for: Maternal biomarker patterns for metabolism and inflammation in pregnancy are influenced by multiple micronutrient supplementation and associated with child biomarker patterns and nutritional status at 9-12 years of age
Source: PLoS One. 2020 Aug 7;15(8):e0216848. doi: 10.1371/journal.pone.0216848 (PMC7413500; doi:10.1371/journal.pone.0216848)

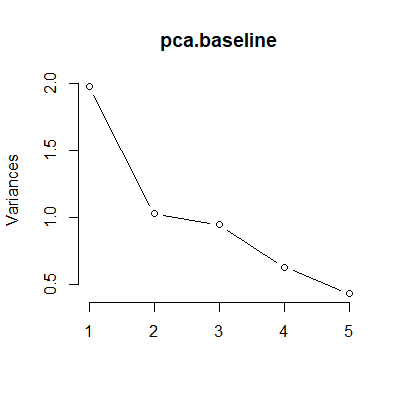


**S1 Figure Screeplot of maternal baseline PCA.**

Supplement: S1 Fig — (DOCX) [file pone.0216848.s002.docx]

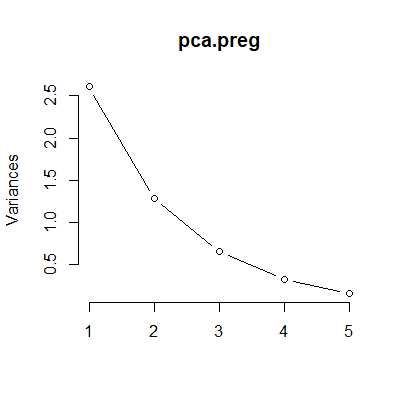


**S2 Figure Screeplot of maternal post-supplementation during prengancy PCA.**

Supplement: S2 Fig — (DOCX) [file pone.0216848.s003.docx]

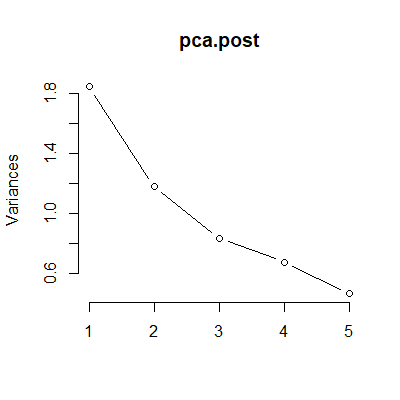


**S3 Figure Screeplot of maternal post-supplementation at post-partum PCA.**

Supplement: S3 Fig — (DOCX) [file pone.0216848.s004.docx]

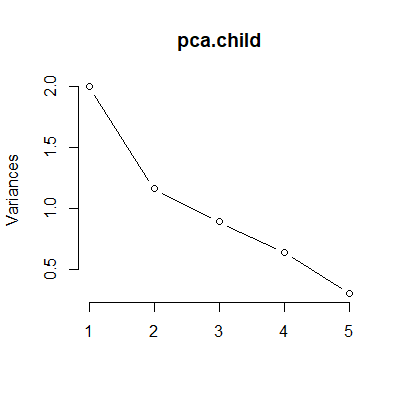


**S4 Figure Screeplot of children PCA.**

Supplement: S4 Fig — (DOCX) [file pone.0216848.s005.docx]

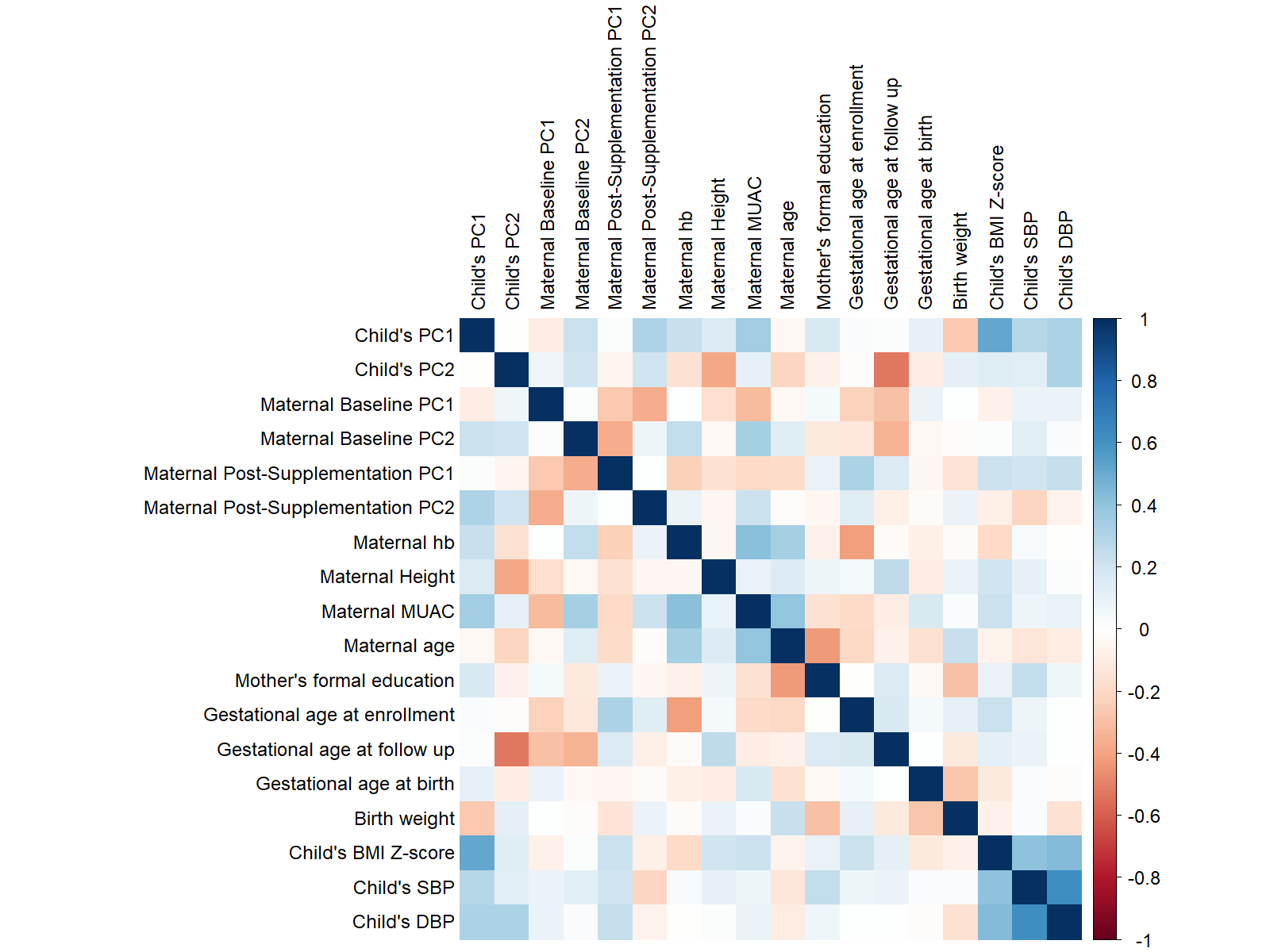


**S6 Figure Correlation map between principle components and all variables.**

Supplement: S6 Fig — (DOCX) [file pone.0216848.s007.docx]
