# Supplementary material for: Maternal biomarker patterns for metabolism and inflammation in pregnancy are influenced by multiple micronutrient supplementation and associated with child biomarker patterns and nutritional status at 9-12 years of age
Source: PLoS One. 2020 Aug 7;15(8):e0216848. doi: 10.1371/journal.pone.0216848 (PMC7413500; doi:10.1371/journal.pone.0216848)
Supplement: S5 Fig — Cross validation was performed using ‘mdatools’ package. Blue line: cumulative variance of PCA result. Red line: cumulative variance of cross validation result. (DOCX) [file pone.0216848.s006.docx]

**
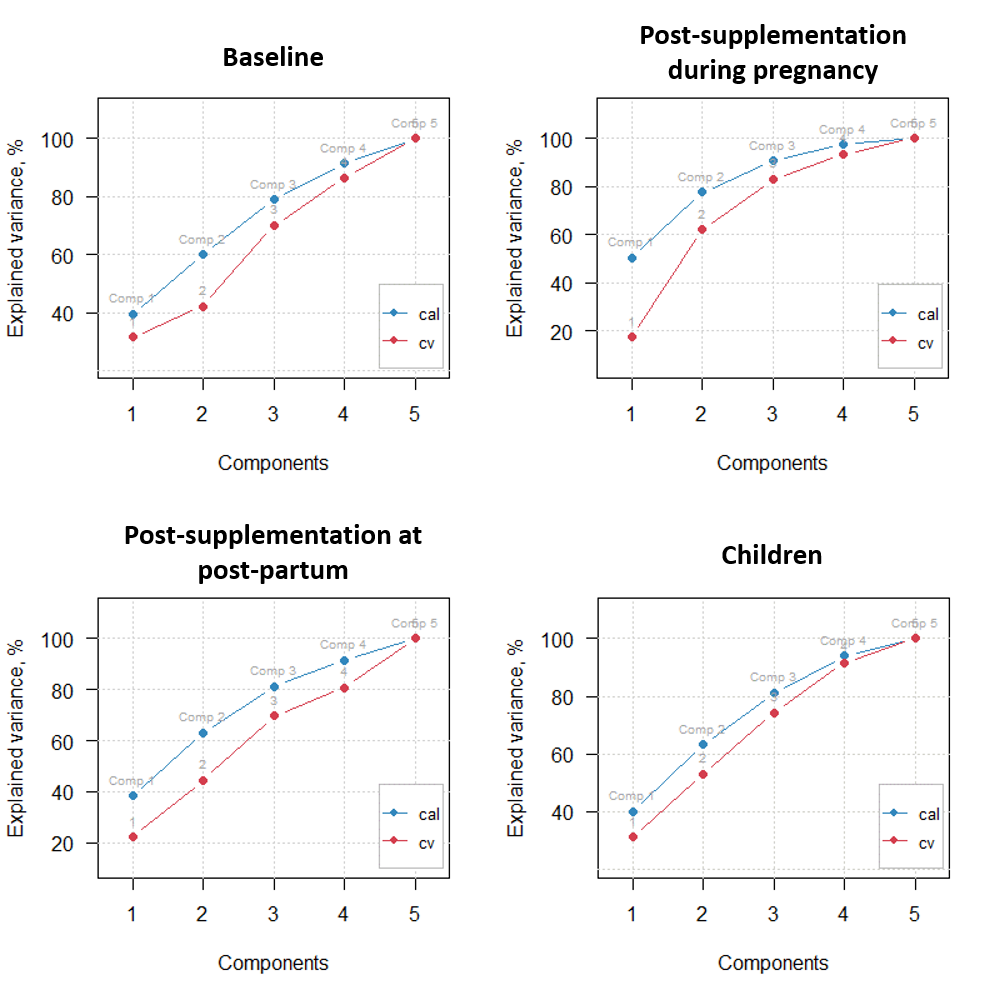
**

**S5 Figure Cross validation of cumulative variance.**

Cross validation was performed using ‘mdatools’ package. Blue line: cumulative variance of PCA result. Red line: cumulative variance of cross validation result.
