# Supplementary material for: Maternal biomarker patterns for metabolism and inflammation in pregnancy are influenced by multiple micronutrient supplementation and associated with child biomarker patterns and nutritional status at 9-12 years of age
Source: PLoS One. 2020 Aug 7;15(8):e0216848. doi: 10.1371/journal.pone.0216848 (PMC7413500; doi:10.1371/journal.pone.0216848)
Supplement: S1 Table — (DOCX) [file pone.0216848.s008.docx]

**S1 Table. Biomarker concentrations of pregnant women during baseline, post-supplementation during pregnancy, post-supplementation at post-partum, and in children**

| Biomarker | Baseline  (n=44) | | | Post-supplementation during pregnancy  (n=18) | | | Post-supplementation at post-partum (N=26) | | | Children  (n=44) | | |
| --- | --- | --- | --- | --- | --- | --- | --- | --- | --- | --- | --- | --- |
|  | IFA  (n=22) | MMN  (n=22) | p-value | IFA  (n=9) | MMN  (n=9) | p-value | IFA  (n=13) | MMN  (n=13) | p-value | IFA  (n=22) | MMN  (n=22) | p-value |
| VDBP (µg/mL) | 46.6  (29.2 - 72.1) | 58.0  (36.0 - 90.5) | 0.203 | 25.9  (18.6 - 37.5) | 40.1  (33.6 - 53.2) | 0.171 | 74.6  (29.3 - 110.6) | 37.8  (29.7 - 44.9) | 0.264 | 18.3  (12.8 - 24.2) | 19.9  (17.3 - 24.7) | 0.302 |
| Adiponectin (µg/mL) | 2.8 ± 1.2 | 3.4 ± 1.4 | 0.121† | 2.3  (2.0 - 2.5) | 2.8  (2.6 - 3.2) | 0.113 | 3.4  (2.1 - 4.3) | 3.2  (2.4 - 3.8) | 0.724 | 5.5 ± 1.4 | 6.0 ± 2.2 | 0.435† |
| RBP4 (µg/mL) | 25.5  (22.8 - 35.2) | 32.8  (21.7 - 37.2) | 0.357 | 17.2  (14.1 - 20.7) | 29.7  (18.3 - 36.3) | 0.136 | 40.3 ± 17.4 | 38.9 ± 10.3 | 0.804† | 25.2  (20.0 - 29.2) | 23.5  (18.9 - 27.6) | 0.825 |
| CRP (µg/mL) | 1.9  (1.2 - 3.0) | 2.1  (0.4 - 3.7) | 0.807 | 1.3  (0.4 - 1.5) | 1.2  (1.0 - 2.4) | 0.730 | 0.9  (0.4 - 1.2) | 0.2  (0.1 - 0.9) | 0.153 | 0.2  (0.1 - 0.7) | 0.2  (0.1 - 0.4) | 0.370 |
| Leptin (ng/mL) | 8.7  (4.8 - 12.5) | 7.6  (5.9 - 14.6) | 0.719 | 16.4  (7.6 - 19.0) | 12.2  (10.7 - 24.9) | 0.796 | 4.8  (1.9 - 5.9) | 3.2  (2.2 - 4.4) | 0.687 | 3.6  (2.3 - 5.0) | 3.0  (2.5 - 6.1) | 0.935 |

Normally distributed variables were presented as mean (±standard deviation). Non-normally distributed variables were presented as median (interquartile range). VDBP: vitamin D binding protein; RBP4: retinol binding protein 4; CRP: C-reactive protein. Significant *p* values <0.05. †Normal distributed variables were compared using Student T-Test.
