# Supplementary material for: Maternal biomarker patterns for metabolism and inflammation in pregnancy are influenced by multiple micronutrient supplementation and associated with child biomarker patterns and nutritional status at 9-12 years of age
Source: PLoS One. 2020 Aug 7;15(8):e0216848. doi: 10.1371/journal.pone.0216848 (PMC7413500; doi:10.1371/journal.pone.0216848)
Supplement: S5 Table — (DOCX) [file pone.0216848.s012.docx]

**S5 Table. Principal component analysis results of children’s biomarkers**

|  | PC1 | PC2 | PC3 | PC4 | PC5 |
| --- | --- | --- | --- | --- | --- |
| Eigenvalues | 1.997 | 1.163 | 0.892 | 0.644 | 0.303 |
| % variance accounted for | 39.950 | 23.268 | 17.841 | 12.872 | 6.069 |
| Loadings |  |  |  |  |  |
| Log VDBP | 0.464 | -0.529 | 0.349 | 0.233 | -0.573 |
| Log Adiponectin | 0.157 | 0.609 | 0.732 | -0.248 | -0.089 |
| Log RBP4 | 0.600 | 0.086 | 0.041 | 0.485 | 0.630 |
| Log CRP | 0.497 | -0.226 | -0.167 | -0.800 | 0.184 |
| Log Leptin | 0.391 | 0.540 | -0.559 | 0.096 | -0.484 |

PC: principal component. VDBP: vitamin D binding protein. RBP4: retinol binding protein. CRP: C-reactive protein. Principal component analysis (PCA) was performed to reduce the five biomarkers that accounted for most of the variance. PC1 and PC2 were retained as described in Materials and Methods; i.e. meeting least two of three criteria including eigenvalue cutoffs defined by Horn’s parallel analysis, being robust to outlier prediction based on the squared residual distance Q and Hotelling T^2^ distance and pattern of variance explained, and frequency of associations in regression analyses that exceeds what would be expected as assessed by the Fisher Exact test.
