# Supplementary material for: Maternal biomarker patterns for metabolism and inflammation in pregnancy are influenced by multiple micronutrient supplementation and associated with child biomarker patterns and nutritional status at 9-12 years of age
Source: PLoS One. 2020 Aug 7;15(8):e0216848. doi: 10.1371/journal.pone.0216848 (PMC7413500; doi:10.1371/journal.pone.0216848)
Supplement: S6 Table — (DOCX) [file pone.0216848.s013.docx]

**S6 Table. Association between maternal biomarkers at baseline and maternal nutritional status**

|  | Maternal Biomarkers at Baseline | | | | | | | | | | | | | | | | | | | | | | | | |
| --- | --- | --- | --- | --- | --- | --- | --- | --- | --- | --- | --- | --- | --- | --- | --- | --- | --- | --- | --- | --- | --- | --- | --- | --- | --- |
|  | Log VDBP (n=44) (17.8 ± 0.61) | | | | | Log Adiponectin (n=44) (14.8 ± 0.48) | | | | | Log RBP4 (n=44) (17.1 ± 0.48) | | | | | Log CRP (n=44) (14.3 ± 1.33) | | | | | Log Leptin (n=44) (9.1 ± 0.78) | | | | |
|  | Unadjusted | | Adjusted | | | Unadjusted | | Adjusted | | | Unadjusted | | Adjusted | | | Unadjusted | | Adjusted | | | Unadjusted | | Adjusted | | |
|  | B | *p* | B | ꞵ | *p* | B | *p* | B | ꞵ | *p* | B | *p* | B | ꞵ | *p* | B | *p* | B | ꞵ | *p* | B | *p* | B | ꞵ | *p* |
| Hb at baseline (g/dL) | -0.109 | 0.105 | -0.084 | -0.138 | 0.29 | 0.031 | 0.52 | 0.006 | 0.013 | 0.931 | 0.043 | 0.429 | 0.018 | 0.038 | 0.751 | -0.303 | 0.06 | -0.07 | -0.053 | 0.686 | 0.137 | 0.188 | 0.034 | 0.044 | 0.721 |
| Height (cm) | -0.004 | 0.885 | -0.011 | -0.018 | 0.703 | 0.008 | 0.691 | 0.013 | 0.027 | 0.587 | 0.027 | 0.243 | 0.031 | 0.065 | 0.143 | 0.038 | 0.583 | 0.051 | 0.038 | 0.412 | -0.045 | 0.318 | -0.009 | -0.012 | 0.792 |
| MUAC (mm) | 0.00021 | 0.954 | 0.003 | 0.005 | 0.385 | 0.003 | 0.28 | 0.003 | 0.006 | 0.321 | 0.004 | 0.205 | 0.007 | 0.015 | **0.013** | 0.003 | 0.746 | 0.004 | 0.003 | 0.658 | 0.011 | **0.036** | 0.012 | 0.015 | **0.012** |
| Gestational age (weeks) | 0.028 | **0.027** | 0.024 | 0.039 | 0.092 | 0.011 | 0.234 | 0.004 | 0.008 | 0.712 | -0.008 | 0.455 | 0.005 | 0.010 | 0.655 | 0.052 | 0.087 | 0.052 | 0.039 | 0.092 | -0.011 | 0.577 | 0.029 | 0.037 | 0.094 |

VDBP: vitamin D binding protein; RBP4: retinol binding protein 4; CRP: C-reactive protein; B: unstandardized beta coefficient; ꞵ: standardized beta coefficient by divided B with SD of log biomarkers; Hb: hemoglobin; MUAC: mid-upper arm circumference. Association analyses were performed using unadjusted and adjusted linear models. For adjusted regressions, the dependent variables were baseline maternal biomarkers and the independent variables were maternal Hb at baseline, maternal height, maternal MUAC at baseline, and gestational age at enrolment. Significant *p* values <0.05.
