# Supplementary material for: Maternal biomarker patterns for metabolism and inflammation in pregnancy are influenced by multiple micronutrient supplementation and associated with child biomarker patterns and nutritional status at 9-12 years of age
Source: PLoS One. 2020 Aug 7;15(8):e0216848. doi: 10.1371/journal.pone.0216848 (PMC7413500; doi:10.1371/journal.pone.0216848)
Supplement: S7 Table — (DOCX) [file pone.0216848.s014.docx]

**S7 Table. Association between maternal biomarkers at baseline and post-supplementation**

|  | Maternal Biomarkers at post-supplementation | | | | | | | | | | | | | | | | | | | | | | | | |
| --- | --- | --- | --- | --- | --- | --- | --- | --- | --- | --- | --- | --- | --- | --- | --- | --- | --- | --- | --- | --- | --- | --- | --- | --- | --- |
|  | Log VDBP (n=44) (17.6 ± 0.74) | | | | | Log Adiponectin (n=44) (14.8 ± 0.44) | | | | | Log RBP4 (n=44) (17.2 ± 0.49) | | | | | Log CRP (n=44) (13.5 ± 1.47) | | | | | Log Leptin (n=44) (8.7 ± 0.94) | | | | |
|  | Unadjusted | | Adjusted | | | Unadjusted | | Adjusted | | | Unadjusted | | Adjusted | | | Unadjusted | | Adjusted | | | Unadjusted | | Adjusted | | |
|  | B | *p* | B | ꞵ | *p* | B | *p* | B | ꞵ | *p* | B | *p* | B | ꞵ | *p* | B | *p* | B | ꞵ | *p* | B | *p* | B | ꞵ | *p* |
| Baseline Log VDBP | 0.089 | 0.618 | -0.142 | -0.192 | 0.475 | 0.068 | 0.537 | -0.017 | -0.039 | 0.856 | 0.023 | 0.85 | -0.135 | -0.276 | 0.227 | 0.137 | 0.713 | -0.074 | -0.050 | 0.847 | -0.013 | 0.957 | 0.257 | 0.273 | 0.116 |
| Baseline Log Adiponectin | 0.069 | 0.765 | -0.135 | -0.182 | 0.624 | 0.601 | **<0.001** | 0.545 | 1.239 | **<0.001** | -0.023 | 0.884 | -0.336 | -0.686 | **0.034** | 0.198 | 0.676 | -0.51 | -0.347 | 0.338 | -0.249 | 0.41 | -0.252 | -0.268 | 0.261 |
| Baseline RBP4 | 0.568 | 0.009 | 0.547 | 0.739 | 0.105 | 0.183 | 0.19 | -0.332 | -0.755 | **0.042** | 0.507 | **0.001** | 0.429 | 0.876 | **0.025** | -0.136 | 0.776 | 0.244 | 0.166 | 0.702 | -0.24 | 0.432 | -0.207 | -0.220 | 0.441 |
| Baseline CRP | 0.017 | 0.842 | 0.008 | 0.011 | 0.93 | 0.134 | **0.006** | 0.045 | 0.102 | 0.309 | -0.006 | 0.913 | 0.023 | 0.047 | 0.664 | 0.54 | **0.001** | 0.61 | 0.415 | **0.002** | -0.116 | 0.291 | -0.03 | -0.032 | 0.687 |
| Baseline Leptin | 0.073 | 0.597 | 0.051 | 0.069 | 0.759 | 0.09 | 0.294 | 0.109 | 0.248 | 0.173 | -0.08 | 0.411 | -0.048 | -0.098 | 0.6 | 0.325 | 0.262 | 0.073 | 0.050 | 0.819 | 0.759 | **<0.001** | 0.527 | 0.561 | **<0.001** |
| Hb at baseline | -0.058 | 0.454 | -0.133 | -0.180 | 0.141 | 0.031 | 0.52 | 0.001 | 0.002 | 0.973 | 0.043 | 0.429 | -0.033 | -0.067 | 0.511 | -0.303 | 0.06 | -0.226 | -0.154 | 0.194 | 0.137 | 0.188 | 0.156 | 0.166 | **0.036** |
| Height (cm) | 0.028 | 0.375 | -0.004 | -0.005 | 0.915 | 0.008 | 0.691 | -0.012 | -0.027 | 0.469 | 0.027 | 0.243 | -0.017 | -0.035 | 0.396 | 0.038 | 0.583 | 0.03 | 0.020 | 0.659 | -0.045 | 0.318 | 0.013 | 0.014 | 0.653 |
| MUAC (cm) | 0.006 | 0.171 | 0.004 | 0.005 | 0.401 | 0.003 | 0.28 | 0.004 | 0.009 | 0.166 | 0.004 | 0.205 | 0.005 | 0.010 | 0.13 | 0.003 | 0.746 | 0.004 | 0.003 | 0.707 | 0.011 | **0.036** | 0.002 | 0.002 | 0.702 |
| MMN supplementation | 0.000 | 1.000 | -0.129 | -0.174 | 0.577 | 0.133 | 0.317 | 0.104 | 0.236 | 0.351 | 0.136 | 0.364 | 0.232 | 0.473 | 0.079 | -0.205 | 0.649 | -0.035 | -0.024 | 0.938 | -0.027 | 0.926 | -0.077 | -0.082 | 0.68 |
| Timing post-supplementation at pregnancy | -0.396 | 0.095 | -0.336 | -0.454 | 0.24 | -0.249 | 0.061 | -0.378 | -0.859 | **0.008** | -0.561 | **<0.001** | -0.505 | -1.031 | **0.003** | 0.756 | 0.094 | 0.92 | 0.626 | 0.098 | 1.365 | **<0.001** | 1.005 | 1.069 | **<0.001** |
| Interaction model: |  |  |  |  |  |  |  |  |  |  |  |  |  |  |  |  |  |  |  |  |  |  |  |  |  |
| Baseline Log VDBP*supp |  |  | 0.203 | 0.274 | 0.617 |  |  | -0.065 | -0.148 | 0.742 |  |  | -0.082 | -0.167 | 0.724 |  |  | -0.927 | -0.631 | 0.242 |  |  | 0.49 | 0.521 | 0.138 |
| Baseline Log Adiponectin*supp |  |  | 0.451 | 0.609 | 0.359 |  |  | 0.131 | 0.298 | 0.552 |  |  | 0.216 | 0.441 | 0.4 |  |  | -0.395 | -0.269 | 0.657 |  |  | 0.176 | 0.187 | 0.638 |
| Baseline RBP4*supp |  |  | -0.751 | -1.015 | 0.146 |  |  | -0.063 | -0.143 | 0.782 |  |  | -0.157 | -0.320 | 0.556 |  |  | -1.707 | -1.161 | 0.057 |  |  | -0.428 | -0.455 | 0.264 |
| Baseline CRP*supp |  |  | 0.127 | 0.172 | 0.456 |  |  | 0.166 | 0.377 | **0.04** |  |  | 0.13 | 0.265 | 0.18 |  |  | -0.084 | -0.057 | 0.804 |  |  | 0.026 | 0.028 | 0.853 |
| Baseline Leptin*supp |  |  | 0.127 | 0.172 | 0.456 |  |  | 0.063 | 0.143 | 0.658 |  |  | 0.1 | 0.204 | 0.548 |  |  | -0.323 | -0.220 | 0.576 |  |  | -0.415 | -0.441 | 0.08 |

VDBP: vitamin D binding protein; RBP4: retinol binding protein 4; CRP: C-reactive protein; B: unstandardized beta coefficient; ꞵ: standardized beta coefficient by divided B with SD of log biomarkers; Hb: hemoglobin; MUAC: mid-upper arm circumference; MMN: multiple micronutrients. Analysis were performed using unadjusted and adjusted linear models. For adjusted regressions, the dependent variables were post-supplementation maternal biomarkers, and the independent variables were baseline maternal biomarkers, maternal Hb at baseline, maternal height, maternal MUAC at baseline, and MMN/IFA supplementation. The model used for interaction (*) included baseline maternal biomarkers (VDBP/adiponectin/RBP4/CRP/leptin)*MMN/IFA supplementation. †: regression using Rfit. Significant *p* values <0.05.
