# Supplementary material for: Maternal biomarker patterns for metabolism and inflammation in pregnancy are influenced by multiple micronutrient supplementation and associated with child biomarker patterns and nutritional status at 9-12 years of age
Source: PLoS One. 2020 Aug 7;15(8):e0216848. doi: 10.1371/journal.pone.0216848 (PMC7413500; doi:10.1371/journal.pone.0216848)
Supplement: S8 Table — (DOCX) [file pone.0216848.s015.docx]

**S8 Table. Association between child biomarkers and maternal biomarkers at baseline**

|  | Child Biomarkers | | | | | | | | | | | | | | | | | | | |
| --- | --- | --- | --- | --- | --- | --- | --- | --- | --- | --- | --- | --- | --- | --- | --- | --- | --- | --- | --- | --- |
|  | Log VDBP (n=44) | | | | Log Adiponectin (n=44) | | | | Log RBP4 (n=44) | | | | Log CRP (n=44) | | | | Log Leptin (n=44) | | | |
|  | Unadjusted | | Adjusted | | Unadjusted | | Adjusted | | Unadjusted | | Adjusted | | Unadjusted | | Adjusted | | Unadjusted | | Adjusted | |
|  | B | *p* | B | *p* | B | *p* | B | *p* | B | *p* | B | *p* | B | *p* | B | *p* | B | *p* | B | *p* |
| Baseline Log VDBP | -0.13 | 0.25 | -0.086 | 0.465 | -0.045 | 0.563 | 0.016 | 0.855 | 0.014 | 0.872 | 0.042 | 0.683 | -0.154 | 0.622 | -0.037 | 0.91 | 0.314 | 0.111 | 0.385 | 0.07 |
| Baseline Log Adiponectin | 0.005 | 0.975 | -0.128 | 0.425 | 0.039 | 0.698 | 0.179 | 0.129 | 0.026 | 0.806 | 0.029 | 0.834 | 0.038 | 0.925 | -0.431 | 0.332 | -0.107 | 0.672 | -0.053 | 0.851 |
| Baseline RBP4 | 0.245 | 0.088 | 0.222 | 0.206 | -0.119 | 0.233 | -0.324 | **0.014** | 0.108 | 0.313 | 0.035 | 0.815 | 0.261 | 0.514 | -0.045 | 0.925 | -0.041 | 0.872 | -0.446 | 0.152 |
| Baseline CRP | -0.006 | 0.907 | -0.001 | 0.991 | -0.044 | 0.222 | -0.06 | 0.132 | -0.031 | 0.42 | -0.037 | 0.424 | 0.198 | 0.165 | 0.212 | 0.16 | -0.090 | 0.325 | -0.112 | 0.243 |
| Baseline Leptin | 0.043 | 0.625 | 0.011 | 0.901 | 0.105 | 0.082 | 0.099 | 0.121 | 0.040 | 0.538 | 0.016 | 0.832 | 0.201 | 0.41 | -0.056 | 0.816 | 0.311 | **0.042** | 0.24 | 0.123 |
| Hb at baseline (g/dL) | 0.11 | **0.024** | 0.084 | 0.128 | 0.031 | 0.362 | 0.015 | 0.698 | 0.049 | 0.184 | 0.048 | 0.31 | 0.097 | 0.48 | -0.076 | 0.609 | -0.074 | 0.399 | -0.135 | 0.162 |
| Height (cm) | 0.06 | **0.003** | 0.056 | **0.008** | -0.006 | 0.683 | 0.003 | 0.849 | 0.007 | 0.668 | 0.011 | 0.52 | 0.049 | 0.405 | 0.038 | 0.496 | -0.051 | 0.175 | -0.035 | 0.326 |
| MUAC (cm) | 0.003 | 0.21 | 1.1x10^-4^ | 0.971 | 0.003 | 0.113 | 0.003 | 0.163 | 0.001 | 0.447 | -2.8x10^-4^ | 0.913 | 0.017 | **0.019** | 0.022 | **0.011** | 0.008 | 0.063 | 0.012 | **0.023** |
| Birth weight (kg) | -0.031 | 0.801 | -0.09 | 0.437 | 0.101 | 0.239 | 0.115 | 0.178 | -0.171 | 0.058 | -0.169 | 0.099 | -0.756 | **0.023** | -0.944 | **0.006** | -0.183 | 0.4 | -0.172 | 0.403 |
| Gender: Boy | 0.181 | 0.185 | 0.183 | 0.183 | -0.027 | 0.78 | -0.074 | 0.452 | -0.078 | 0.444 | -0.034 | 0.769 | 0.253 | 0.504 | 0.692 | 0.072 | -0.343 | 0.152 | -0.074 | 0.758 |

VDBP: vitamin D binding protein; RBP4: retinol binding protein 4; CRP: C-reactive protein; post-supp: post-supplementation; B: coefficient of regression; Hb: hemoglobin; MUAC: mid-upper arm circumference; MMN: multiple micronutrients. Analysis was performed using unadjusted and adjusted linear models. For adjusted regressions, the dependent variables were child biomarkers, and the independent variables were baseline maternal biomarkers, maternal Hb at baseline, maternal height, maternal MUAC at baseline, birth weight, and child's gender (boy/girl). Significant *p* values <0.05.
