# Supplementary material for: Maternal biomarker patterns for metabolism and inflammation in pregnancy are influenced by multiple micronutrient supplementation and associated with child biomarker patterns and nutritional status at 9-12 years of age
Source: PLoS One. 2020 Aug 7;15(8):e0216848. doi: 10.1371/journal.pone.0216848 (PMC7413500; doi:10.1371/journal.pone.0216848)
Supplement: S9 Table — (DOCX) [file pone.0216848.s016.docx]

**S9 Table. Association between child biomarkers and maternal biomarkers at post-supplementation**

|  | Child Biomarkers | | | | | | | | | | | | | | | | | | | |
| --- | --- | --- | --- | --- | --- | --- | --- | --- | --- | --- | --- | --- | --- | --- | --- | --- | --- | --- | --- | --- |
|  | Log VDBP (n=44) | | | | Log Adiponectin (n=44) | | | | Log RBP4 (n=44) | | | | Log CRP (n=44) | | | | Log Leptin (n=44) | | | |
|  | Unadjusted | | Adjusted | | Unadjusted | | Adjusted | | Unadjusted | | Adjusted | | Unadjusted | | Adjusted | | Unadjusted | | Adjusted | |
|  | B | *p* | B | *p* | B | *p* | B | *p* | B | *p* | B | *p* | B | *p* | B | *p* | B | *p* | B | *p* |
| Post-supp Log VDBP | 0.116 | 0.211 | -0.038 | 0.728 | -0.125 | **0.05** | -0.079 | 0.336 | 0.073 | 0.289 | 0.083 | 0.364 | 0.452 | 0.074 | 0.232 | 0.456 | 0.228 | 0.162 | 0.448 | **0.031** |
| Post-supp Log Adiponectin | 0.181 | 0.256 | -0.005 | 0.975 | -0.042 | 0.705 | 0.081 | 0.47 | 0.094 | 0.424 | 0.038 | 0.762 | 0.608 | 0.163 | 0.514 | 0.235 | 0.117 | 0.678 | 0.215 | 0.44 |
| Post-supp RBP4 | 0.328 | **0.017** | 0.238 | 0.223 | -0.209 | **0.028** | -0.033 | 0.818 | 0.095 | 0.365 | -0.128 | 0.428 | 0.470 | 0.224 | 0.301 | 0.582 | -0.144 | 0.563 | -0.411 | 0.25 |
| Post-supp CRP | 0.032 | 0.493 | 0.049 | 0.282 | -0.004 | 0.898 | -0.018 | 0.589 | -0.027 | 0.434 | -0.018 | 0.634 | 0.208 | 0.105 | 0.129 | 0.316 | 0.049 | 0.552 | -0.001 | 0.991 |
| Post-supp Leptin | -0.054 | 0.46 | 0.141 | 0.134 | 0.151 | **0.002** | 0.096 | 0.172 | 0.045 | 0.411 | 0.189 | **0.019** | 0.110 | 0.587 | 0.241 | 0.363 | 0.328 | **0.008** | 0.279 | 0.109 |
| Height (cm) | 0.06 | **0.003** | 0.044 | **0.036** | -0.006 | 0.683 | 0.006 | 0.697 | 0.007 | 0.668 | 0.002 | 0.908 | 0.049 | 0.405 | 0.065 | 0.259 | -0.051 | 0.175 | -0.043 | 0.253 |
| Birth weight (kg) | -0.031 | 0.801 | -0.089 | 0.435 | 0.101 | 0.239 | 0.105 | 0.216 | -0.171 | 0.058 | -0.183 | 0.058 | -0.756 | **0.023** | -0.816 | **0.015** | -0.183 | 0.400 | -0.092 | 0.658 |
| Gender: Boy | 0.181 | 0.185 | 0.287 | **0.033** | -0.027 | 0.78 | -0.137 | 0.161 | -0.078 | 0.444 | 0.033 | 0.758 | 0.253 | 0.504 | 0.641 | 0.088 | -0.343 | 0.152 | -0.36 | 0.137 |
| MMN supplementation | 0.127 | 0.355 | 0.017 | 0.892 | 0.043 | 0.652 | 0.033 | 0.73 | 0.007 | 0.943 | 0.034 | 0.753 | -0.541 | 0.149 | -0.71 | 0.058 | -0.043 | 0.858 | 0.135 | 0.57 |
| Timing post-supplementation at pregnancy | -0.305 | **0.025** | -0.411 | 0.065 | 0.257 | **0.006** | 0.145 | 0.37 | -0.120 | 0.243 | -0.386 | **0.038** | 0.007 | 0.986 | -0.027 | 0.965 | 0.490 | **0.041** | 0.151 | 0.704 |

VDBP: vitamin D binding protein; RBP4: retinol binding protein 4; CRP: C-reactive protein; post-supp: post-supplementation; B: coefficient of regression; Hb: hemoglobin; MUAC: mid-upper arm circumference; MMN: multiple micronutrients. Analysis was performed using unadjusted and adjusted linear models. For adjusted regressions, the dependent variables were child biomarkers, and the independent variables were post-supplementation maternal biomarkers, maternal Hb at baseline, maternal height, maternal MUAC at baseline, birth weight, and child's gender (boy/girl). Significant *p* values <0.05.
