# Supplementary material for: Maternal biomarker patterns for metabolism and inflammation in pregnancy are influenced by multiple micronutrient supplementation and associated with child biomarker patterns and nutritional status at 9-12 years of age
Source: PLoS One. 2020 Aug 7;15(8):e0216848. doi: 10.1371/journal.pone.0216848 (PMC7413500; doi:10.1371/journal.pone.0216848)
Supplement: S10 Table — (DOCX) [file pone.0216848.s017.docx]

**S10 Table. Association between child's outcome and maternal biomarkers at baseline**

|  | Child's outcome | | | | | | | | | | | |
| --- | --- | --- | --- | --- | --- | --- | --- | --- | --- | --- | --- | --- |
|  | BMIZ (n=44) | | | | SBP (n=43) | | | | DBP (n=43) | | | |
|  | Unadjusted | | Adjusted | | Unadjusted | | Adjusted | | Unadjusted | | Adjusted | |
|  | B | *p* | B | *p* | B | *p* | B | *p* | B | *p* | B | *p* |
| Baseline Log VDBP | 0.339 | 0.192 | 0.334 | 0.262 | 0.956 | 0.708 | 0.97 | 0.741 | 0.54 | 0.786 | 1.346 | 0.536 |
| Baseline Log Adiponectin | 0.071 | 0.830 | 0.166 | 0.679 | 0.365 | 0.91 | 3.874 | 0.327 | 0.556 | 0.824 | 3.925 | 0.183 |
| Baseline RBP4 | 0.204 | 0.544 | -0.302 | 0.492 | -0.062 | 0.985 | -1.722 | 0.694 | -3.277 | 0.199 | -6.871 | **0.04** |
| Baseline CRP | -0.053 | 0.660 | -0.164 | 0.231 | -2.196 | 0.059 | -2.679 | 0.056 | -0.907 | 0.324 | -1.175 | 0.249 |
| Baseline Leptin | -0.011 | 0.959 | -0.178 | 0.418 | -1.41 | 0.476 | -1.47 | 0.495 | -0.031 | 0.984 | 0.127 | 0.936 |
| Hb at baseline (g/dL) | -0.155 | 0.176 | -0.259 | 0.064 | 0.273 | 0.807 | 0.671 | 0.631 | -0.042 | 0.962 | 0.741 | 0.475 |
| Height (cm) | 0.061 | 0.210 | 0.067 | 0.196 | 0.314 | 0.509 | 0.277 | 0.586 | 0.042 | 0.909 | 0.16 | 0.672 |
| MUAC (cm) | 0.009 | 0.125 | 0.018 | **0.021** | 0.03 | 0.612 | -0.002 | 0.984 | 0.027 | 0.558 | 0.016 | 0.788 |
| Birth weight (kg) | -0.175 | 0.540 | -0.31 | 0.294 | 0.4759 | 0.864 | 0.553 | 0.848 | -2.179 | 0.309 | -1.409 | 0.512 |
| Gender: Boy | -0.277 | 0.381 | 0.091 | 0.791 | -1.077 | 0.728 | -0.903 | 0.786 | -2.988 | 0.211 | -2.807 | 0.26 |
| Child BMIZ |  |  |  |  | 4.064 | **0.007** | 3.843 | **0.036** | 3.444 | **0.003** | 3.118 | **0.022** |

VDBP: vitamin D binding protein; RBP4: retinol binding protein 4; CRP: C-reactive protein; post-supp: post-supplementation; B: coefficient of regression; Hb: hemoglobin; MUAC: mid-upper arm circumference; MMN: multiple micronutrients; BMI: body mass index; SBP: systolic blood pressure; DBP: diastolic blood pressure. Analysis was performed using unadjusted and adjusted linear models. For adjusted regressions, the dependent variables were BMIZ, SBP, DBP, and the independent variables were baseline maternal biomarkers, maternal Hb at baseline, maternal height, maternal MUAC at baseline, birth weight, child's gender (boy/girl), MMN/IFA supplementation, and child BMIZ for models with SBP and DBP as dependent variables. Significant *p* values <0.05.
