# Supplementary material for: Maternal biomarker patterns for metabolism and inflammation in pregnancy are influenced by multiple micronutrient supplementation and associated with child biomarker patterns and nutritional status at 9-12 years of age
Source: PLoS One. 2020 Aug 7;15(8):e0216848. doi: 10.1371/journal.pone.0216848 (PMC7413500; doi:10.1371/journal.pone.0216848)
Supplement: S11 Table — (DOCX) [file pone.0216848.s018.docx]

**S11 Table. Association between child's outcome and maternal biomarkers at post-supplementation**

|  | Child's outcome | | | | | | | | | | | |
| --- | --- | --- | --- | --- | --- | --- | --- | --- | --- | --- | --- | --- |
|  | BMIZ (n=44) | | | | SBP (n=43) | | | | DBP (n=43) | | | |
|  | Unadjusted | | Adjusted | | Unadjusted | | Adjusted | | Unadjusted | | Adjusted | |
|  | B | *p* | B | *p* | B | *p* | B | *p* | B | *p* | B | *p* |
| Post-supp Log VDBP | 0.311 | 0.146 | 0.356 | 0.257 | 2.243 | 0.284 | 2.535 | 0.381 | 1.523 | 0.35 | 1.652 | 0.442 |
| Post-supp Log Adiponectin | 0.273 | 0.459 | 0.22 | 0.609 | 0.741 | 0.836 | -0.893 | 0.821 | 2.791 | 0.312 | 3.053 | 0.304 |
| Post-supp RBP4 | 0.233 | 0.476 | -0.357 | 0.515 | 1.311 | 0.681 | -1.143 | 0.82 | -0.516 | 0.835 | -2.157 | 0.564 |
| Post-supp CRP | -0.020 | 0.853 | -0.063 | 0.625 | -0.367 | 0.73 | -0.57 | 0.625 | 0.082 | 0.921 | -0.366 | 0.673 |
| Post-supp Leptin | -0.192 | 0.256 | -0.242 | 0.362 | -0.669 | 0.685 | -1.171 | 0.631 | 0.752 | 0.558 | -0.098 | 0.957 |
| Height (cm) | 0.061 | 0.210 | 0.057 | 0.322 | 0.314 | 0.509 | -0.015 | 0.977 | 0.042 | 0.909 | 0.005 | 0.989 |
| Birth weight (kg) | -0.175 | 0.540 | -0.146 | 0.649 | 0.4759 | 0.864 | 1.105 | 0.706 | -2.179 | 0.309 | -1.231 | 0.572 |
| Gender: Boy | -0.277 | 0.381 | -0.209 | 0.572 | -1.077 | 0.728 | -1.254 | 0.709 | -2.988 | 0.211 | -2.846 | 0.26 |
| MMN supplementation | 0.132 | 0.678 | 0.124 | 0.735 | 5.637 | 0.063 | 5.212 | 0.129 | 1.592 | 0.508 | 1.35 | 0.591 |
| Timing post-supplementation at pregnancy | -0.254 | 0.431 | 0.292 | 0.636 | 0.548 | 0.863 | 3.605 | 0.525 | 2.572 | 0.294 | 4.418 | 0.298 |
| Child BMIZ |  |  |  |  | 4.064 | **0.007** | 3.427 | **0.049** | 3.444 | **0.003** | 2.947 | **0.025** |

VDBP: vitamin D binding protein; RBP4: retinol binding protein 4; CRP: C-reactive protein; post-supp: post-supplementation; B: coefficient of regression; Hb: hemoglobin; MUAC: mid-upper arm circumference; MMN: multiple micronutrients; BMI: body mass index; SBP: systolic blood pressure; DBP: diastolic blood pressure. Analysis was performed using unadjusted and adjusted linear models. For adjusted regressions, the dependent variables were BMIZ, SBP, DBP, and the independent variables were post-supplementation maternal biomarkers, maternal Hb at baseline, maternal height, maternal MUAC at baseline, birth weight, child's gender (boy/girl), MMN/IFA supplementation, and child BMIZ for models with SBP and DBP as dependent variables. Significant *p* values <0.05.
