# Supplementary material for: Maternal biomarker patterns for metabolism and inflammation in pregnancy are influenced by multiple micronutrient supplementation and associated with child biomarker patterns and nutritional status at 9-12 years of age
Source: PLoS One. 2020 Aug 7;15(8):e0216848. doi: 10.1371/journal.pone.0216848 (PMC7413500; doi:10.1371/journal.pone.0216848)
Supplement: S13 Table — (DOCX) [file pone.0216848.s020.docx]

**S13 Table. Spearman correlation of maternal biomarkers at baseline and post-supplementation during pregnancy**

|  | Baseline  VDBP | Post-supp  VDBP | Baseline  Adiponectin | Post-supp  Adiponectin | Baseline  RBP4 | Post-supp  RBP4 | Baseline  CRP | Post-supp  CRP | Baseline  Leptin | Post-supp  Leptin |
| --- | --- | --- | --- | --- | --- | --- | --- | --- | --- | --- |
| Baseline  VDBP | 1 | 0.26 | 0.46 | 0.37 | 0.42 | 0.11 | -0.08 | -0.29 | -0.17 | 0.02 |
| Post-supp  VDBP | 0.26 | 1 | 0.40 | 0.63** | 0.30 | 0.81** | 0.23 | 0.28 | 0.20 | -0.28 |
| Baseline  Adiponectin | 0.46 | 0.4 | 1 | 0.77*** | 0.74** | 0.23 | 0.49* | 0.10 | 0.60** | 0.06 |
| Post-supp  Adiponectin | 0.37 | 0.63** | 0.77*** | 1 | 0.45 | 0.50* | 0.49* | 0.14 | 0.41 | -0.04 |
| Baseline  RBP4 | 0.42 | 0.30 | 0.74 | 0.45 | 1 | 0.14 | 0.30 | 0.03 | 0.38 | -0.02 |
| Post-supp  RBP4 | 0.11 | 0.81*** | 0.23 | 0.50** | 0.14 | 1 | 0.10 | 0.11 | 0.03 | -0.21 |
| Baseline  CRP | -0.08 | 0.23 | 0.49** | 0.49* | 0.30 | 0.10 | 1 | 0.71** | 0.28 | -0.27 |
| Post-supp  CRP | -0.29 | 0.28 | 0.10 | 0.14 | 0.03 | 0.11 | 0.71* | 1 | 0.16 | -0.26 |
| Baseline  Leptin | -0.17 | 0.20 | 0.60 | 0.41 | 0.38 | 0.03 | 0.28 | 0.16 | 1 | 0.50* |
| Post-supp  Leptin | 0.02 | -0.28 | 0.06 | -0.04 | -0.02 | -0.21 | -0.27 | -0.26 | 0.50* | 1 |

VDBP: vitamin D binding protein; RBP4: retinol binding protein 4; CRP: C-reactive protein. *** correlation is significant at the level 0.001. ** correlation is significant at the level 0.01. * correlation is significant at the level 0.05.
