# Supplementary material for: Maternal biomarker patterns for metabolism and inflammation in pregnancy are influenced by multiple micronutrient supplementation and associated with child biomarker patterns and nutritional status at 9-12 years of age
Source: PLoS One. 2020 Aug 7;15(8):e0216848. doi: 10.1371/journal.pone.0216848 (PMC7413500; doi:10.1371/journal.pone.0216848)
Supplement: S14 Table — (DOCX) [file pone.0216848.s021.docx]

**S14 Table. Spearman correlation of maternal biomarkers at baseline and post-supplementation at post-partum**

|  | Baseline  VDBP | Post-supp  VDBP | Baseline  Adiponectin | Post-supp  Adiponectin | Baseline  RBP4 | Post-supp  RBP4 | Baseline  CRP | Post-supp  CRP | Baseline  Leptin | Post-supp  Leptin |
| --- | --- | --- | --- | --- | --- | --- | --- | --- | --- | --- |
| Baseline  VDBP | 1 | -0.19 | 0.03 | 0.03 | 0.16 | -0.25 | 0.34 | 0.35 | 0.36 | 0.27 |
| Post-supp  VDBP | -0.19 | 1 | -0.18 | -0.10 | 0.42* | 0.51** | -0.16 | -0.08 | 0.18 | 0.31 |
| Baseline  Adiponectin | 0.03 | -0.18 | 1 | 0.79*** | -0.02 | -0.37 | 0.46* | 0.14 | -0.17 | -0.17 |
| Post-supp  Adiponectin | 0.03 | -0.10 | 0.79*** | 1 | -0.01 | -0.28 | 0.43* | 0.23 | -0.07 | 0 |
| Baseline  RBP4 | 0.16 | 0.42* | -0.02 | -0.01 | 1 | 0.57** | -0.35 | -0.04 | 0.55** | 0.45** |
| Post-supp  RBP4 | -0.25 | 0.51** | -0.37 | -0.28 | 0.57** | 1 | -0.35 | -0.1 | 0.05 | 0.2 |
| Baseline  CRP | 0.34 | -0.16 | 0.46* | 0.43* | -0.35 | -0.35 | 1 | 0.39* | -0.1 | -0.1 |
| Post-supp  CRP | 0.35 | -0.08 | 0.14 | 0.23 | -0.04 | -0.10 | 0.39* | 1 | 0.01 | 0.14 |
| Baseline  Leptin | 0.36 | 0.18 | -0.17 | -0.07 | 0.55** | 0.05 | -0.1 | 0.01 | 1 | 0.62** |
| Post-supp  Leptin | 0.27 | 0.31 | -0.17 | 0 | 0.45* | 0.2 | -0.1 | 0.14 | 0.62 | 1 |

VDBP: vitamin D binding protein; RBP4: retinol binding protein 4; CRP: C-reactive protein. *** correlation is significant at the level 0.001. ** correlation is significant at the level 0.01. * correlation is significant at the level 0.05.
